# BaRDIC: robust peak calling for RNA–DNA interaction data

**DOI:** 10.1093/nargab/lqae054

**Published:** 2024-05-20

**Authors:** Dmitry E Mylarshchikov, Arina I Nikolskaya, Olesja D Bogomaz, Anastasia A Zharikova, Andrey A Mironov

**Affiliations:** Faculty of Bioengineering and Bioinformatics, Lomonosov Moscow State University, Leninskiye Gory, Moscow 119234, Russia; Faculty of Bioengineering and Bioinformatics, Lomonosov Moscow State University, Leninskiye Gory, Moscow 119234, Russia; Faculty of Bioengineering and Bioinformatics, Lomonosov Moscow State University, Leninskiye Gory, Moscow 119234, Russia; Faculty of Bioengineering and Bioinformatics, Lomonosov Moscow State University, Leninskiye Gory, Moscow 119234, Russia; Kharkevich Institute for Information Transmission Problems RAS, Bolshoy Karetny per., Moscow 127051, Russia; Faculty of Bioengineering and Bioinformatics, Lomonosov Moscow State University, Leninskiye Gory, Moscow 119234, Russia; Kharkevich Institute for Information Transmission Problems RAS, Bolshoy Karetny per., Moscow 127051, Russia

## Abstract

Chromatin-associated non-coding RNAs play important roles in various cellular processes by targeting genomic loci. Two types of genome-wide NGS experiments exist to detect such targets: ‘one-to-all’, which focuses on targets of a single RNA, and ‘all-to-all’, which captures targets of all RNAs in a sample. As with many NGS experiments, they are prone to biases and noise, so it becomes essential to detect ‘peaks’—specific interactions of an RNA with genomic targets. Here, we present BaRDIC—Binomial RNA–DNA Interaction Caller—a tailored method to detect peaks in both types of RNA–DNA interaction data. BaRDIC is the first tool to simultaneously take into account the two most prominent biases in the data: chromatin heterogeneity and distance-dependent decay of interaction frequency. Since RNAs differ in their interaction preferences, BaRDIC adapts peak sizes according to the abundances and contact patterns of individual RNAs. These features enable BaRDIC to make more robust predictions than currently applied peak-calling algorithms and better handle the characteristic sparsity of all-to-all data. The BaRDIC package is freely available at https://github.com/dmitrymyl/BaRDIC.

## Introduction

Chromatin-associated non-coding RNAs play important roles in various cellular processes (1–3). Many sequencing techniques are now available to localize RNAs on the whole chromatin, which can be broadly classified into ‘one-to-all’ (OTA) and ‘all-to-all’ (ATA) methods (4,5). OTA methods (CHART-seq (6), RAP (7), ChIRP-seq (8), ChOP-seq (9)) hybridize biotinylated RNA-specific oligonucleotide probes to find the contacts of a single target RNA. High-throughput ATA methods (GRID-seq (10), ChAR-seq (11), MARGI (12), iMARGI (13), RADICL-seq (14), RedC (15)) detect genome-wide contacts of all potential chromatin-associated RNAs using RNA–DNA proximity ligation. Identifying specific interactions of an individual RNA with a particular genomic location, a process called ‘peak-calling’, has been a focus of all RNA–DNA interaction studies. However, RNA–DNA interaction data contain biological and technology-specific biases, which hinder the detection of real RNA-binding sites on the chromatin. Similar to peak-calling algorithms in ChIP-seq data and chromatin loop detection in Hi-C data, there is a need to develop tailored methods for identifying specific interactions in RNA–DNA interaction data.

Specific interaction loci can be defined as genomic regions where the number of contacts is significantly higher than expected in a given background model. Ideally, the background model captures all possible sources of biases. One of these biases is chromatin heterogeneity, which includes chromatin accessibility, amplification bias, and copy number variation. This bias is also present in ChIP-seq datasets. And since the nature of OTA data is also similar to that of ChIP-seq data due to common experimental steps, a ChIP-seq specific peak caller MACS2 is usually applied to OTA data, using an input track, which evaluates chromatin heterogeneity. For ATA data, chromatin heterogeneity can be inferred from the dataset itself, according to GRID data processing protocol (10). The endogenous background here is based on *trans* chromosomal interactions of mRNAs, assuming such contacts are mostly non-specific. In contrast, RADICL protocol (14) proposed a uniform binomial model that ignores the heterogeneous nature of the chromatin.

**Figure 1.**
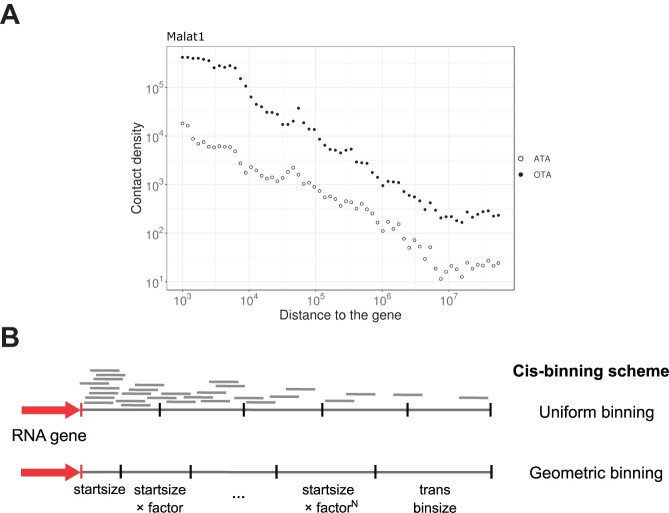
(**A**) Dependency of contact density on the distance between the RNA source gene and chromatin target loci (scaling) in double logarithmic coordinates for Malat1. Black points: ChIRP OTA data on mESC (18), white points: ATA GRID data on mESC (10). (**B**) *Cis* binning strategy with geometrically increasing bins provides more even contact coverage in the vicinity of the source gene.

Another source of bias is the decay of contact density with increasing distance from the RNA transcription site. This effect was described in several studies dedicated to RNA–DNA interaction data analysis (14,15). We observed this distance-dependent effect in both OTA and ATA datasets (Figure 1A) with our previously developed RNA-Chrom database (16). Analogous to Hi-C, we will refer to this phenomenon as ‘RD-scaling’ (17). MACS2, GRID and RADICL approaches do not consider RD-scaling in their models, therefore, these algorithms might overestimate the statistical significance of contacts neighbouring an RNA source gene.

Finally, functionally diverse classes of RNAs have different patterns of genome occupancy. For instance, an enhancer RNA – promoter interaction may exhibit a narrow peak profile, while a small nuclear RNA can appear to be broadly distributed along gene bodies. Furthermore, we expect that peak width would be affected by the amount of the data, which refers to resolution. In ATA datasets, RNAs differ in contact frequency, while limited sequencing depth leads to data sparsity as a result of incomplete contact capture. Consequently, there is a ground to introduce a variable peak size for different RNAs depending on their abundances and distribution patterns along the chromatin. Existing GRID and RADICL procedures propose fixed-size bins for all RNAs, which can reduce the resolution for RNAs with narrow binding sites, and lower the statistical power for RNAs with more diffuse binding sites.

In this work, we introduce a versatile tool BaRDIC (Binomial RNA–DNA Interaction Caller), that utilizes a binomial model to identify genomic regions significantly enriched in RNA-chromatin interactions, or ‘peaks’, in ATA and OTA data. Our approach combines strategies from Hi-C and ChIP-seq data analysis to account for several key features of RNA–DNA interaction data:

- chromatin heterogeneity;
- RD-scaling;
- data sparsity and distinct RNA contact patterns.

To evaluate the performance of BaRDIC peak calling, we established a simulation framework for RNA–DNA contact data with known peak locations. Since there is no gold standard for real data, we evaluated BaRDIC performance on the simulated data and real datasets by considering the biological principles of RNA–chromatin interactions. Based on our assessment, BaRDIC yields accurate predictions of RNA–DNA interactions from both OTA and ATA data.

## Materials and methods

### Simulations of RNA–DNA contact data

To obtain ground-truth data about specific RNA–DNA interactions, we established a simulation framework for RNA–DNA contact data. In this framework, contacts of only one RNA on two chromosomes, ‘*cis*’ and ‘*trans*’, are simulated. Chromatin heterogeneity is modelled with Poisson distribution while RD-scaling—with a sigmoid function. The combination of the two is used to sample coordinates of non-specific contacts. Coordinates of peak summits are also sampled from this combination, while coordinates of corresponding specific contacts are distributed normally around peak summits. The procedure is described in more detail in the Supplementary Note II. The simulation framework is implemented as part of the BaRDIC package (see Results).

To obtain quality metrics of BaRDIC peak calling, we defined True Positives (TP) as the number of BaRDIC peaks overlapping simulated peak summits, False Positives (FP) as the number of BaRDIC peaks not overlapping simulated peak summits, and False Negatives (FN) as the number of simulated peak summits not overlapping BaRDIC peaks. Then we calculated two values:


\begin{eqnarray*} \mathrm{Precision} = \frac{\mathrm{TP}}{\mathrm{TP + FP}},\ \mathrm{Recall} = \frac{\mathrm{TP}}{\mathrm{TP + FP}} \end{eqnarray*}

and constructed Precision–Recall curves by varying the global q-value threshold of peaks. We calculated the area-under-the-curve (AUC) for each replicate using the scikit-learn package, took their average and calculated the 95% confidence interval for it. To plot the Precision–Recall curve, we pooled all the values from the replicates and estimated the trend line with mean and the 95% confidence interval with bootstrapping, both of which were done with seaborn.lineplot.

### Peak-calling on OTA data

Pre-processed contacts and MACS2 peaks for OTA experiments were extracted from the RNA-Chrom database (16). The data identifiers (Exp. ID) are as follows: 94 (RAP-RNA Malat1), 102 (ChIRP Halr1) for mESC cell line, 90 (CHART Paupar) for mouse neuroblastoma cell line N2A. Details on the experiments and data processing protocol are described in the RNA-Chrom database.

Background track for OTA experiments was constructed from input data and converted to begGraph format in 1kb bins using bedtools package (19). The type of background used by BaRDIC was set to a pre-constructed background track (-bt custom). Due to relatively high resolution of OTA experiments, the following parameters were provided for peak-calling on OTA data: 400 nt minimum *trans* bin size (–trans_min), 100 nt initial *cis* bin size (–cis_start 100), 50 nt step to find the optimal *trans* bin size (–trans_step). For Halr1 (Haunt), the peaks for the retinoic acid-induced mESC sample were used for analysis as the peaks from the control mESC sample do not intersect HOXA cluster genes.

### Peak-calling on ATA data

Contacts for the GRID-seq ATA experiment on the mESC cell line (Exp. ID 6) and RNA gene annotation for the mouse genome (except X-RNA biotype) were extracted from the RNA-Chrom database. Peaks were called with default parameters. Peaks by RADICL procedure were called according to the algorithm description from the original study (14). Since we did not succeed in reproducing the original GRID peak-calling method, the peaks published by authors were used in analysis (10). To compare BaRDIC with the GRID peak caller, the publicly available peaks were converted from mm9 to mm10 using LiftOver (20) (only 0.86% of peaks were lost after the conversion). It is worth noting that the peak comparison may be skewed due to differences in the data processing protocol of the GRID-seq authors and our group.

## Results

### BaRDIC algorithm

The BaRDIC algorithm consists of three steps:

1. Binning: for each RNA, chromosomes are partitioned into non-overlapping genomic intervals (bins), and the number of contacts is calculated within bins.
2. Statistical modelling: for each RNA, parameters of the background model are calculated in every bin. *P*-values are calculated based on them.
3. Multiple testing correction with Benjamini–Hochberg procedure (21).

To increase the statistical power and speed up the algorithm’s performance, only RNAs with >1000 contacts are selected by default. Steps 1 and 2 are atomic with regard to individual RNAs, so we describe them for one RNA only.

#### Binning

Bin sizes are estimated for each RNA separately. The binning strategy differs for *cis* interactions, which occur on a chromosome harboring the RNA gene, and *trans* interactions—with other chromosomes.

In the case of random ligation, we expect that *trans* contacts are distributed uniformly along chromosomes. Therefore, we apply a uniform binning strategy for *trans* interactions.

For binning in *cis*, RD-scaling must be taken into account. Also, we assume long-range *cis* interactions are similar to *trans* interactions, analogous to observations in Hi-C data analysis (22). In our *cis* binning strategy, chromosomes are partitioned into non-uniform bins of size increasing in a geometric progression from the source gene. *Cis* bin size increases until it exceeds the *trans* bin size; all subsequent *cis* bins are uniform and equal to the *trans* bin in size (see Figure 1B):


\begin{eqnarray*} cis\ \mathrm{binsize}(i) = \min (\mathrm{startsize} \cdot \mathrm{factor}^i,\ trans\ \mathrm{binsize}) \end{eqnarray*}

To find optimal bin sizes, we adapted RSEG (23) and JAMM (24) approaches that were developed for ChIP-seq peak-calling. This approach minimizes a cost function that is dependent on the bin size and distribution of contact counts for each bin size, thus striking a balance between resolution and sufficient bin coverage. Details of this procedure are described in the Supplementary Note I section 1.3.

#### Statistical modelling

To model the background distribution of RNA–DNA contacts in bins, we introduce a frequentist model similar to those used in ChIP-seq and Hi-C data analysis (25,26). Assuming that contacts arising from random binding are independent, we consider the number of contacts *X*_*ij*_ of RNA *i* in bin *j* to be binomially distributed:


\begin{eqnarray*} X_{ij}\sim Bin\left( N_{i}, p_{ij} \right), \end{eqnarray*}

where *N*_*i*_ is the total number of contacts produced by RNA *i* (except the gene body), *p*_*ij*_ is the background probability.

We assume that the number of observed contacts *O*_*ij*_ is a realization of the random variable *X*_*ij*_. Statistical estimation comes down to inferring the only model parameter *p*_*ij*_ from the observed data.

For *trans* bins, only chromatin heterogeneity plays a role. In ATA experiments, we estimate the parameter of the background model by counting mRNA *trans* contacts as proposed in the GRID procedure. The background probability of a single contact of RNA *i* to appear in the *j*-th *trans* bin is assumed to be as follows:


\begin{eqnarray*} \hat{p}_{ij} = \hat{p}^{bg}_{j}=\frac{N^{bg}_{j}}{N^{bg}}, \end{eqnarray*}

where $N^{bg}_{j}$ is the number of contacts from the background in a bin *j*, *N*^*bg*^ is the total number of background contacts. For OTA experiments, we use contacts from the input sample similar to ChIP-seq analysis.

For *cis* bins, we additionally consider RD-scaling. To do that, we define a scaling factor *f*(*d*_*ij*_) that depends on the distance between the source gene of RNA *i* and bin *j*: $\hat{p}_{ij} = f(d_{ij}) \cdot \hat{p}^{bg}_{ij}$. We estimate the scaling factor as $f\left( d_{ij} \right) = \frac{O_{ij}/N_{i}}{\hat{p}_{j}^{bg}}$ and smooth its profile using a cubic smoothing spline in double logarithmic coordinates. As $\hat{f}\left( d_{ij} \right)$ values vary substantially for different RNAs, splines are calculated for every RNA separately. To exclude putative *cis* peaks from the background model, we perform a two-step scaling estimation procedure similar to Fit-Hi-C (25) and HiC-DC (27), removing bins with statistically high contact coverage. We observed RD-scaling factors at large distances (More than 10Mb) to approach the value of 1 (Supplementary Figure S2), which indicates that distal RNA–DNA *cis* contacts behave just like *trans* contacts, which is unlike in Hi-C data (28,29), where distal *cis* interactions are subject to increased noise compared to proximal *cis* interactions. This is due to biological differences between DNA–DNA and RNA–DNA contacts – while specific long-range DNA–DNA *cis* interactions are unlikely, specific RNA–DNA interactions can be at any distance from the RNA source gene.

To hold $\sum _{j}^{}\hat{p}_{ij} = 1$, we renormalize probability estimates for each RNA *i*. As a result, the sum of background probabilities for *cis* bins equals the fraction of *cis* contacts of each RNA, the same is true for *trans* bins.

We expect that a specific binding event results in a high enrichment of contacts relative to the background. The resulting *P*-value is then computed with the right-sided binomial test using estimated background parameters. *P*-values from non-zero bins are subjected to the multiple testing correction using the Benjamini–Hochberg procedure, which is applied in two ways: for all RNAs simultaneously producing global *q*-values and for each RNA separately producing RNA *q*-values. By default, peaks are selected based on a threshold for global *q*-values.

#### Implementation

The algorithm is implemented as a package for Python 3 (30) with a command line interface and is available at https://github.com/dmitrymyl/BaRDIC. It is based on numpy (31), pandas, scipy (32) and statsmodels packages (33). The bioframe package (34) is used for operations with genomic intervals. Since binning and statistical evaluation are performed for each RNA separately, these steps are parallelized. To understand how different factors influence the peak calling, users can specify to not model the background (it will be treated as a uniform one) and/or not to model the RD-scaling (all scaling factors will be equal to 1).

The input and output data are provided in standardized BED and narrowPeak file formats, which makes BaRDIC easy to use and simple to perform downstream analyses on its results. To organize the data storage and speed up the data access, we developed two HDF5-based data formats (35) for internal operations, which were inspired by the cool (36) format. Detailed algorithm description and format schemas together with sensible values of parameters are given in Supplementary Note I.

### Overview of evaluation and use cases

Evaluating the peak caller performance on RNA–DNA interaction data is still an open problem, given the lack of a gold standard or a ‘true’ dataset. We established a simulation framework for RNA–DNA contact data with known peak locations, that models both chromatin heterogeneity and RD-scaling (see Materials and methods). To obtain results on real data with no known peak locations, we assessed peak calling results based on biological principles of RNA–chromatin interactions:

We assessed the quality of BaRDIC peaks based on well-studied RNAs with known binding preferences;
We compared BaRDIC performance on the real data with the three current approaches to identify RNA-binding sites — MACS2 peak caller for OTA and original methods from ATA studies, GRID-seq and RADICL-seq, hereafter referred to as GRID-peak and RADICL-peak.

Importantly, these three current approaches (MACS2, GRID-peak, RADICL-peak) do not model all typical biases in RNA–DNA interaction data, e.g. RD-scaling, so none of them can be considered the gold standard.

### BaRDIC performance on simulated data

**Figure 2.**
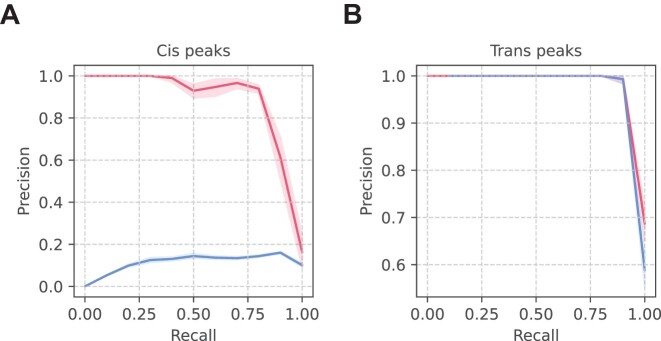
Precision–Recall curves derived from *cis* and *trans* peak-calling results of BaRDIC on 20 simulated RNA–DNA datasets by varying the q-value threshold. Trend lines are averages with shaded areas representing 95% confidence intervals. Colours denote the BaRDIC peak-calling mode, default (pink) or RD-scaling-inactivated (blue). (**A**) Area-under-the-curve (AUC) values for *cis* peak-calling: default BaRDIC 0.862 (CI 95% 0.818, 0.906), BaRDIC without RD-scaling correction 0.168 (CI 95% 0.142, 0.193). (**B**) AUC values for *trans* peak-calling: default BaRDIC 0.998 (CI 95% 0.995, 1), BaRDIC without RD-scaling correction 0.998 (CI 95% 0.995, 1).

To assess how BaRDIC recovers true peak locations, we simulated RNA–DNA contact data (see Materials and methods), called peaks from it, and calculated Precision and Recall metrics while varying the q-value threshold. To produce robust estimates, we ran simulations 20 times and calculated average Precision–Recall curves. BaRDIC performed exceptionally well achieving both high precision and recall values at most q-value thresholds (Figure 2A, B) both in *cis* and in *trans*.

Next, we wanted to assess how individual effects contribute to the performance of peak calling. Our simulation framework allowed us to reliably evaluate only the contribution of modelling the RD-scaling (see Supplementary Note II section 2.3). BaRDIC with no estimation of RD-scaling performed much poorer in calling *cis* peaks compared to BaRDIC in the default mode (5 times decrease of the area under the Precision-Recall curve), but similarly in calling *trans* peaks (Figure 2A, B), which underlies the importance of taking RD-scaling into account when inferring specific RNA–DNA interactions.

Overall, our simulations showed that BaRDIC performs extremely well in recovering true peak locations in simulated RNA–DNA contact data.

### General assessment of BaRDIC ATA peaks

**Figure 3.**
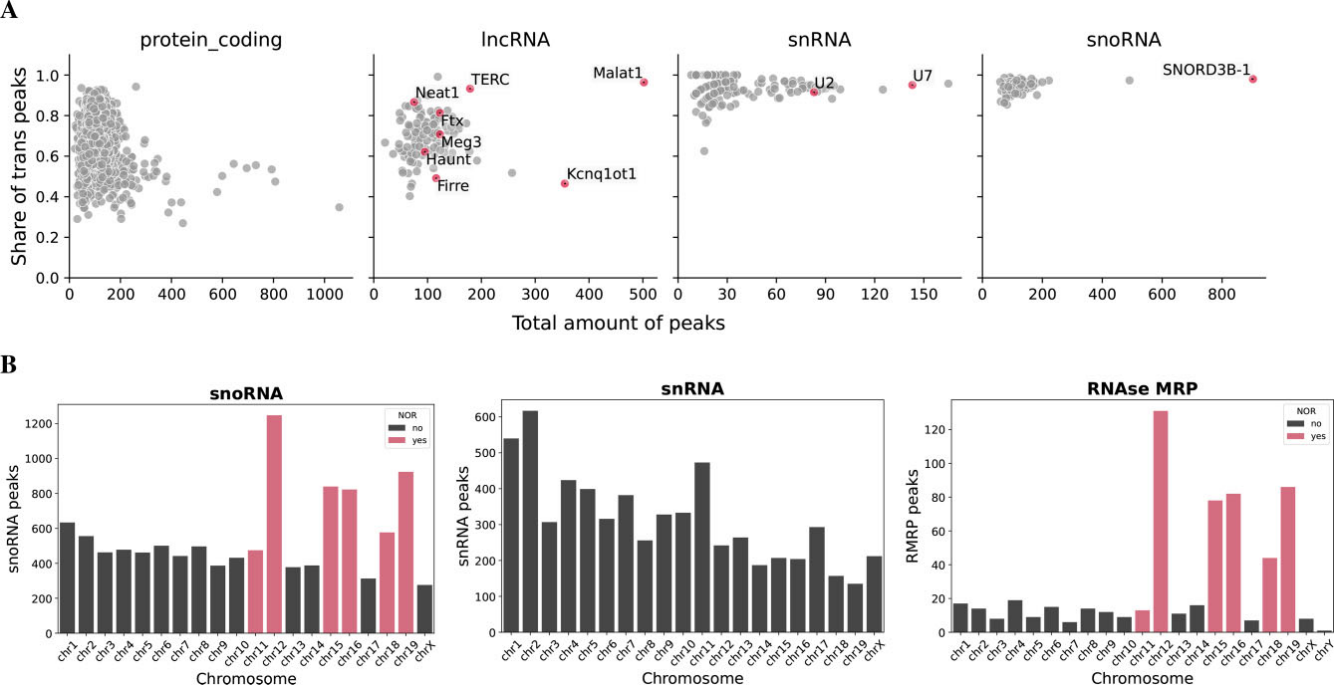
Global assessment of specific RNA interactions identified by BaRDIC for GRID data on mESC data. (**A**) Fractions of *trans* peaks and the number of ATA peaks for select RNA biotypes. (**B**) Distribution of snoRNAs, snRNAs, and MRP RNase peaks across mouse chromosomes. Chromosomes carrying nucleolus organizer regions (NORs) are highlighted in pink.

We evaluated BaRDIC on real data by using GRID-seq dataset obtained on the mESC, a cell line for which many OTA experiments are also available (See Supplementary Tables S1 and S2). Only RNAs with at least 1000 contacts in the ATA experiment were selected for peak calling. In total, BaRDIC identified specific interactions mediated by 7741 unique RNAs in GRID-seq data on mESC (Supplementary Figure S1 and S2). For the majority of RNAs, the size of *trans* bins ranges from 30 to 80 kb, with a median of 50 kb (Supplementary Figure S3). RNAs of distinct biotypes were found to have different preferences for *cis* and *trans* interactions. In general, small non-coding RNAs have a large fraction of *trans* peaks, while long non-coding RNAs can generate both *cis* and *trans* peaks (Figure 3A). Notably, RNA preferences do not correlate with the number of RNA peaks, which suggests BaRDIC is robust against data sparsity.

For further analysis, 1007 unique RNAs were selected: all RNAs with at least one BaRDIC peak, except for ribosomal RNAs and mRNAs. To generally assess specific interactions in ATA data, we focused on RNAs with well-studied binding patterns. As expected, Malat1, SNORD3B-1, TERC, Neat1 and RMRP prefer to contact in trans, while Kcnq1ot1, Firre and Haunt (Halr1) favor *cis* interactions (Figure 3A, Supplementary Figure S4). Long ncRNAs as a class have variable preferences because they can act both as *cis* and *trans* regulators (37). At the same time, most lncRNAs and protein-coding RNAs in general preferred cis interactions based on GRID-peak and RADICL-peak results (Supplementary Figure S5, S6), which highlights differences in peak calling when RD-scaling is taken into account. SnoRNAs generate many *trans* peaks as detected with BaRDIC since as they interact with chromosomes carrying the nucleolus organizer regions (NORs): chromosomes 12, 15, 16, 18, 19 except for chromosome 11 (Figure 3B) (38). A similar distribution is observed for RNase MRP (RMRP) consistent with its localization in the nucleus (39). Apparently, in this particular mESC sample, chromosome 11 does not carry active NOR loci. In contrast, snRNAs do not have specific chromosomal preferences: the number of peaks on chromosomes correlates with their lengths (*r*_*S*_ = 0.71, *P*-value < 3.1e−4).

Taken together, the coincidence of *cis* and *trans* peaks distribution with known RNA binding preferences validates BaRDIC peaks at the coarse-grain level.

### Detailed comparison of peaks of individual RNAs

To assess the quality of BaRDIC peaks at the local level, we obtained peaks for several RNAs with known binding patterns, for which OTA experiments are available (Supplementary Tables S1, S2). When comparing OTA and ATA experiments for a particular RNA, it is crucial to keep in mind that ATA contacts are much sparser (Supplementary Figure S7A) and located at varying distances from defined OTA peaks due to the differences in experimental procedures (Supplementary Figure S8). Therefore, in addition to the direct comparison of peaks, we compare genes intersecting peaks, similar to the RADICL approach (14).

To highlight features of BaRDIC, we chose three non-coding RNAs (ncRNAs) with distinct distributions of contacts. ncRNA Paupar exhibits virtually no RD-scaling; ncRNA Malat1 is abundant in ATA datasets and exhibits a pronounced RD-scaling; ncRNA Halr1 is unabundant RNA in ATA datasets, regulates gene expression in *cis* and therefore should have peaks very close to the source gene.

### Paupar: ncRNA with no RD-scaling

Paupar is a vertebrate-conserved lncRNA whose expression is restricted to the central nervous system. CHART-seq revealed that Paupar is predominantly associated with promoters and 5-UTR regions of protein-coding genes and is almost evenly distributed across chromosomes, except for the lack of contacts on the X chromosome. We showed that the RD-scaling effect is not pronounced for Paupar (Supplementary Figure S9E). Thus, we can directly compare the performance of BaRDIC and other algorithms given the virtually absent influence of RD-scaling. As ATA data for mESC lacks Paupar interactions, we compared only OTA peaks.

**Figure 4.**
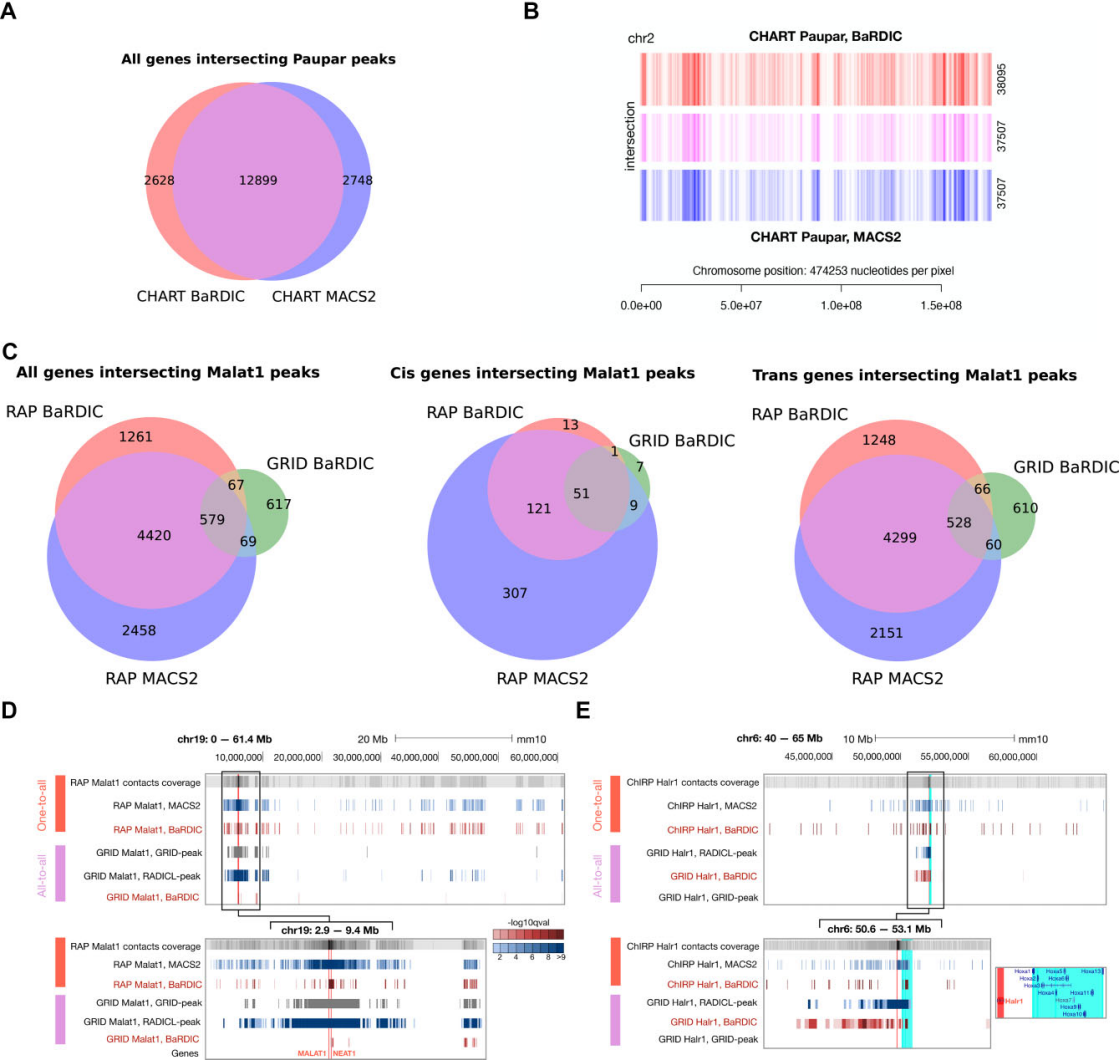
Comparison of peaks obtained by different algorithms for OTA and ATA data on mESC cell line (**A**) Genes intersecting Paupar peaks from CHART data obtained by BaRDIC and MACS2. (**B**) Figure 3 of peak distribution and peaks intersections on ‘parenta’ chromosome 2, CHART data. (**C**) Genes intersecting Malat1 peaks from RAP and GRID mESC data. From left to right: all genes, *cis* genes, *trans* genes. (**D**) Representative genome browser view of Malat1 peaks identified by different algorithms in RAP OTA data (red) and ATA data (purple). Scales are given: the entire length of mouse chromosome 19 and the vicinity of the Malat1 gene on chromosome 19. Vertical red lines represent Malat1 and Neat1 source genes. (**E**) Representative genome browser view of Halr1 peaks identified by different algorithms in ChIRP OTA data (red) and ATA data (purple). The Halr1 gene located on chromosome 6 is highlighted in red, HOXA gene cluster in its vicinity is highlighted in cyan. Tracks are coloured by −log_10_*Q* statistical significance when appropriate.

Sets of genes that intersect BaRDIC peaks and MACS2 peaks for OTA were very similar (Figure 4A). We confirmed spatial co-localization of BaRDIC and MACS2 peaks using GenometriCorr (40) metrics (Figure 4B; Supplementary Figure S10B–D). Moreover, the contact densities of common peaks were much higher than the contact densities of scarce BaRDIC-specific and MACS2-specific peaks (Supplementary Figure S10A). Therefore, for an RNA with a diffuse genome-wide distribution and minor RD-scaling effects, BaRDIC peaks are in agreement with the publicly available results. This proves BaRDIC captures chromatin heterogeneity similarly to other peak calling algorithms.

### Malat1: abundant ncRNA with pronounced RD-scaling

Malat1, an abundant lncRNA, is a global regulator of transcription and pre-mRNA splicing. Malat1 has multiple binding sites in *trans*, predominantly in the bodies of actively transcribed genes (41). Moreover, its possible *cis* regulatory role (42,43) in controlling Neat1 expression has been discussed. Remarkably, Malat1 generates over 527 000 contacts in the selected ATA experiment, being the RNA with the largest number of contacts (Supplementary Table S1; Supplementary Figure S11A). Given a sufficient number of Malat1 *trans* and *cis* contacts in ATA data and the availability of corresponding OTA data, we compared multiple peak calling algorithms and especially the influence of the RD-scaling effect (Supplementary Figure S9A, B) on their performance.

BaRDIC on the OTA data reproduces the set of genes that intersect MACS2 peaks (Figure 4C), in addition, peaks are spatially co-localized (Supplementary Figure S12A–D). Detected peaks and covered genes only slightly changed upon an increase of the background track bin size in OTA data (Supplementary Figure S11B), which suggests the background construction procedure in BaRDIC is reasonable and captures local variability of chromatin accurately. When compared with GRID-peak on the ATA data, BaRDIC shows partial reproducibility of genes intersecting peaks (Supplementary Figure S11D, E), which can be attributed to numerous technical differences between data used to call GRID-peak peaks and BaRDIC peaks (see Materials and methods). However, BaRDIC peaks called from ATA data in *trans* showed similar enrichments with *trans* contacts from OTA data compared to GRID-peak and better enrichments of those compared to RADICL-peak peaks (Supplementary Figure S13A–D). Similarly, based on gene overlaps in ATA data, common BaRDIC and GRID-peak ATA peaks contained higher densities of *trans* contacts than algorithm-specific ones (Supplementary Figure S7B). This suggests sparsity of ATA data is another source of discrepancies between BaRDIC and GRID-peak, so different procedures detect different peak regions with low contact enrichments. Also, BaRDIC peaks had broader enrichments of *trans* contacts compared to GRID-peak peaks in ATA data (Supplementary Figure S7B), which hints that differences in the genome binning strategy also contribute to discrepancies between peak calls. Finally, when comparing OTA and ATA peaks together, genes intersecting ATA peaks called with BaRDIC and GRID-peak showed less reproducibility between themselves compared to genes intersecting OTA peaks called with BaRDIC and MACS2 (Supplementary Figure S11D). Hence, due to sparsity, the ATA experiment is less sensitive to specific contacts of MALAT1 than the OTA experiment. Nonetheless, BaRDIC robustly captures background features of both OTA and ATA data to detect specific RNA–DNA interactions.

We observed a pronounced RD-scaling effect in the MALAT1 contact pattern for both OTA and ATA data (Supplementary Figure S9A, B), so we expected it to influence peak-calling results. In OTA data, BaRDIC *cis* peaks formed a subset of MACS2 *cis* peaks (Supplementary Figure S11C) and they were more evenly distributed along chromosome 19 which harbors the Malat1 source gene compared to MACS2 *cis* peaks (Supplementary Figure S12B). Moreover, there were numerous MACS2-specific *cis* peaks and no BaRDIC-specific *cis* peaks at distances up to 10Mb from the Malat1 source gene, which is the range of distances with pronounced RD-scaling effect (Supplementary Figure S11C). All this supports the assessment that MACS2 does not take into account RD-scaling and overestimates the significance of *cis* peaks close to the Malat1 gene boundaries, which is corrected by the BaRDIC algorithm (Figure 4D). In the ATA data, genes intersecting Malat1 *cis* peaks called with BaRDIC comprised a subset of those intersecting *cis* peaks called with GRID-peak. RADICL-peak and GRID-peak calls in ATA data were clustered near the Malat1 source gene (Figure 4D) with little to no BaRDIC *cis* peaks in this neighbourhood except one at the Neat1 gene. This suggests that BaRDIC filters out likely false *cis* peaks that arose from RD-scaling but preserves previously described specific interactions at the Neat1 locus (41). At the same time, MACS2, RADICL-peak, and GRID-peak do not account for RD-scaling and detect more *cis* peaks and corresponding genes overall which are mostly likely false findings.

Overall, BaRDIC was proven to be robust against local variability of chromatin and sparsity of ATA data. Due to RD-scaling correction, BaRDIC improves previous strategies as it does not overestimate the statistical significance of sites proximal to the RNA source gene. At the same time, BaRDIC preserves consensus target genes, which are likely to be true targets of chromatin-associated RNAs.

### Halr1: *cis*-acting unabundant ncRNA

Long ncRNA Halr1 (HOXA upstream noncoding transcript) regulates the HOXA gene cluster during ESC differentiation (44). In the mouse genome, the HOXA gene cluster is located $\scriptstyle \sim$40 kb downstream of the Halr1 transcription site. Halr1 is an unabundant RNA in ATA experiments, with 1724 contacts (23rd percentile) (Supplementary Table S1; Supplementary Figure S11A) and pronounced RD-scaling (Supplementary Figure S9C, D) in GRID mESC data. We therefore included a Halr1 case study to examine the quality of the BaRDIC algorithm on low-contacting RNAs, which form the vast majority of ATA data.

Peaks called with BaRDIC in OTA and ATA data indeed covered the genes of the HOXA gene cluster (Figure 4E). In contrast, GRID-peak identifies no Halr1 peaks. Additionally, the geometric binning procedure in *cis*, implemented in BaRDIC, increases the resolution near the boundaries of the Halr1 gene (Supplementary Figure S14). This results in a more accurate localization of peaks near the RNA source gene.

Overall, BaRDIC outperforms other peak callers in detecting peaks of RNAs with a small number of contacts in the ATA data. In addition, despite accounting for RD-scaling, BaRDIC finds biologically relevant specific interactions near the RNA source gene.

## Discussion

In this paper, we present BaRDIC, a first-of-its-kind versatile algorithm for identifying specific RNA-chromatin interactions in both ‘one-to-all’ (OTA) and ‘all-to-all’ (ATA) data. BaRDIC takes into account typical biases and other features of RNA–DNA interaction data, including chromatin heterogeneity, RD-scaling, and distinct contact patterns of individual RNAs. In the virtual absence of RD-scaling effects, BaRDIC performs comparably with other algorithms. In the presence of RD-scaling, BaRDIC does not overestimate the statistical significance of findings near the source gene of an individual RNA, unlike previously published approaches. The adaptive bin size selection for each RNA and geometric binning near source genes in ATA data allows for more accurate identification of specific binding sites. In addition, BaRDIC is robust to data sparsity, which is a hallmark of ATA experiments.

Due to the absence of a true dataset of specific RNA–DNA interactions that could be used for benchmarking, we established a lightweight simulation framework for RNA–DNA contact data that models both chromatin heterogeneity and RD-scaling. BaRDIC performed exceptionally well in recovering true peak locations while turning off the estimation of RD-scaling led to poorer performance. Since our simulation framework uses the Poisson distribution with fixed lambda parameter to model chromatin heterogeneity, the simulated background track does not recapitulate all hallmark properties of the real background: inflation of zero counts (telomeres, centromeres, and dropout), as well as overdispersion of counts and the presence of extremely high counts (highly open chromatin and low complexity regions). Due to that, our modelled background is close to uniform (see Supplementary Note II section 2.3) and we noticed almost no difference between BaRDIC with the estimation of the background and BaRDIC with uniform background. So, we could not assess the influence of modelling chromatin heterogeneity, however, we confirmed the great influence of modelling RD-scaling on peak calling performance on RNA–DNA contact data.

We found that when calling peaks in OTA data, BaRDIC and the commonly used MACS2 peak caller incompletely reproduce each other’s results on specific *trans* interactions, even with no apparent influence of RD-scaling. This may be due to different strategies of genome binning: BaRDIC relies on non-overlapping fixed-size bins, while MACS2 determines regions (peak candidates) dynamically. Perhaps the combined approach of binning with offsets in BaRDIC would increase the reproducibility of the results. In addition, the discrepancies may arise from different statistical models—a dynamic Poisson model in MACS2 and a dynamic binomial model in BaRDIC. Finally, BaRDIC peaks for RNAs in this study are wider than MACS2 peaks (Supplementary Table S2). Consequently, BaRDIC bins may include more non-specific interactions, which also decreases the resolution. Therefore, we controlled FDR more strictly for BaRDIC findings compared to MACS2 (Supplementary Table S2). However, it is not our goal to achieve the full reproducibility of results between BaRDIC and MACS2 in OTA data, as there is currently no gold standard for specific RNA interactions. Besides, MACS2 does not account for RD-scaling—the prominent bias in RNA–DNA interaction data, particularly in OTA data. Nevertheless, incomplete, but substantial reproducibility of peaks implies that BaRDIC accounts for chromatin heterogeneity similarly to other algorithms used in this area of research.

For all RNAs studied in this paper, peaks from ATA experiments intersect only a fraction of genes corresponding to OTA experiments, and may as well capture some non-target genes. This may be due to the insufficient sequencing depth in ATA experiments and, as a consequence, dropout. In this process, many true RNA binding sites are lost before the sequencing step, and the remaining nonspecific sites distort the statistical significance of detected peaks. This assumption is supported by the fact that the reproducibility of target genes is better for RNAs with a higher number of contacts in ATA data, such as Malat1. Differences in experimental procedures may also contribute to the inconsistency between OTA and ATA peaks (Supplementary Figure S8). OTA experiments are similar to ChIP-seq with a DNA shearing step that allows a relatively accurate identification of a contact region. In contrast, ATA experiments are similar to Hi-C experiments with DNA restriction and proximity ligation steps, which limit observed contact regions to restriction sites. Collectively, OTA and ATA peaks fundamentally cannot be compared properly, so we do not expect an exact match of called interactions due to the differences and limitations of the two types of experiments.

BaRDIC utilizes the binomial distribution to model the number of RNA–DNA contacts in each genomic bin for each RNA. Because of the sparsity of the data, the binomial model can be approximated by the Poisson model (45), which is a frequently used model for read counts in NGS data analysis (46). We chose the binomial distribution to model contact probabilities instead, but conceptually there is no difference from the common practice in the field. However, binomial and Poisson models are quite simple and might not fully account for all properties of RNA–DNA contact data. One example of such properties is again data sparsity, which might lead to the excess of observed zero counts in genomic bins (47). This property can be taken into account with zero-inflated models (48). Another potential property is the overdispersion of contact counts in genomic bins between replicated experiments (49,50). There is not enough evidence that such property applies to RNA–DNA contact data, but it can be verified with the analysis of replicate datasets. If that is the case, one could utilize the negative binomial distribution to model the overdispersion in a single replicate. Finally, both potential properties can be modelled jointly with a zero-inflated or zero-truncated negative binomial distribution, such as done in Hi-C loop calling (27) and ChIP-seq peak calling (51). BaRDIC can be used as a baseline statistical model to verify if these sophisticated models represent RNA–DNA contact data more faithfully.

In this work, we highlighted the importance of the RD-scaling effect when detecting RNA peaks on chromatin, which has already been around in the field. A recent article on ChAR-seq (52) proposes a generative model for predicting peak-like interactions based on mRNA *trans* contacts, with an account for RD-scaling. However, that model suggests fix-sized bins for all RNAs, while BaRDIC proposes bins of variable size, individually for each RNA. This feature allows BaRDIC to model non-uniform contact coverage and take into account distinct distribution patterns of RNAs along the chromatin.

BaRDIC is not only applicable to pairwise RNA–DNA interaction data but can also be applied to multiway RD-SPRITE (53) data. To achieve this, the multiway data needs to be expanded into virtual pairwise RNA–DNA contacts similar to the strategy proposed for analyzing multiway DNA-DNA interactions obtained with Pore-C (54).

BaRDIC package is an easy-to-use tool since it requires only four files as input: a tab-separated file with chromosome sizes, a BED file with DNA parts of contacts, an RNA gene annotation, and information on the background—a list of background mRNAs or an input track. This simplicity allows a user to parallelize peak calling over multiple RNAs with an ATA dataset or streamline BaRDIC application over multiple isolated datasets. Calling peaks on all available OTA and ATA experiments with RD-scaling correction could help to refine known target genomic loci for each RNA of interest. Collectively, the resulting peaks can be organized in a database, such as RNA-Chrom (16). The unified approach will enable unbiased comparisons of specific RNA–DNA interactions between samples and studies as well as with other high throughput omics data.

Scaling correction during peak calling makes RNA–DNA interaction data reconcilable with specific protein-DNA interactions (ChIP-seq peaks), for which scaling does not exist, and specific DNA-DNA interactions (Hi-C loops), for which scaling is observed and accounted for in dedicated algorithms. This harmonization opens a way for unbiased comparisons of these three types of interactomic data for predicting functions of RNA-chromatin interactions. In particular, these comparisons will lead to predictions about how RNAs bind to their genomic targets: either due to a spatial proximity of corresponding DNA loci or due to recruitment by DNA-binding proteins. In turn, GO and GSEA enrichment analysis of genes interacting with novel chromatin-associated RNAs will create a basis for predicting regulatory functions of RNA-chromatin interactions. Taken together, simultaneously accounting for chromatin heterogeneity and RD-scaling in RNA–DNA interaction data enables unbiased and uncomplicated downstream and comparative analyses.

## Supplementary Material

lqae054_Supplemental_Files

## Data Availability

The BaRDIC package is available at https://github.com/dmitrymyl/BaRDIC and https://zenodo.org/records/10649591.
