# Correction to ‘BaRDIC: robust peak calling for RNA–DNA interaction data’

**DOI:** 10.1093/nargab/lqae080

**Published:** 2024-07-02

This is a correction to: Dmitry E Mylarshchikov, Arina I Nikolskaya, Olesja D Bogomaz, Anastasia A Zharikova, Andrey A Mironov, BaRDIC: robust peak calling for RNA–DNA interaction data, *NAR Genomics and Bioinformatics*, Volume 6, Issue 2, June 2024, lqae054, https://doi.org/10.1093/nargab/lqae054

The following corrections have been made to the article.

In the Abstract, the github link was connecting to an incorrect url and has been updated to https://github.com/dmitrymyl/BaRDIC.
Throughout the text, ‘all-to-al’ and ‘one-to-al’ have been corrected to ‘all-to-all’ and ‘one-to-all’.
In the Introduction of the HTML version of the article, bullet points have been added to:
  chromatin heterogeneity;
  RD-scaling;
  data sparsity and distinct RNA contact patterns.
ORCID iDs have been added to authors Dmitry E. Mylarshchikov and Olesja D. Bogomaz.

The published article has been updated.

