# Supplementary material for: BaRDIC: robust peak calling for RNA–DNA interaction data
Source: NAR Genom Bioinform. 2024 May 20;6(2):lqae054. doi: 10.1093/nargab/lqae054 (PMC11106031; doi:10.1093/nargab/lqae054)
Supplement: lqae054_Supplemental_Files [file lqae054_supplemental_files.zip › NARGAB_2023_163_R1_Suppl_Data_rev2.pdf]

Supplementary Data

RNA	Cell line	Total number of contacts	BaRDIC *trans* bin size, Kb	Amount of BaRDIC peaks	Amount of GRID peaks	Amount of RADICL peaks
Malat1	mESC	527 249	20	502	5042	2515
Halr1	mESC	1724	43	96	0	24

Supplementary Table S1: Number of contacts in ATA GRID data in mESC cell line and the number of peaks called with different methods — BaRDIC, GRID-peak, and RADICL-peak. Selected *trans* bin size for BaRDIC is shown; GRID-peak and RADICL-peak bin sizes are fixed and equal 1 Kb and 25 Kb, respectively.

RNA	OTA experiment	Cell line	Total number of contacts	BaRDIC *trans* bin size, bp	Amount of BaRDIC peaks	Amount of MACS2 peaks	MACS2 median peak size, bp
Malat1	RAP	mESC	14 417 923	900	14 166	15 962	394
Paupar	CHART	N2A	59 369 258	400	78 996	68 833	295
Halr1	ChIRP	mESC	4 772 537	5350*	2 835	2 069	178

Supplementary Table S2: Number of contacts and amount of peaks called with BaRDIC and MACS2 for OTA data. As OTA peaks called with BaRDIC are generally wider than those called with MACS2, we applied more stringent thresholds to select more prominent BaRDIC peaks: $-log10$(q-value)$>$ 4 for Malat1, $-log10$(q-value) $>$ 7 for Paupar and Halr1. *Initial *cis* bin size equals 100 bp, Halr1 preferentially interacts in *cis*.

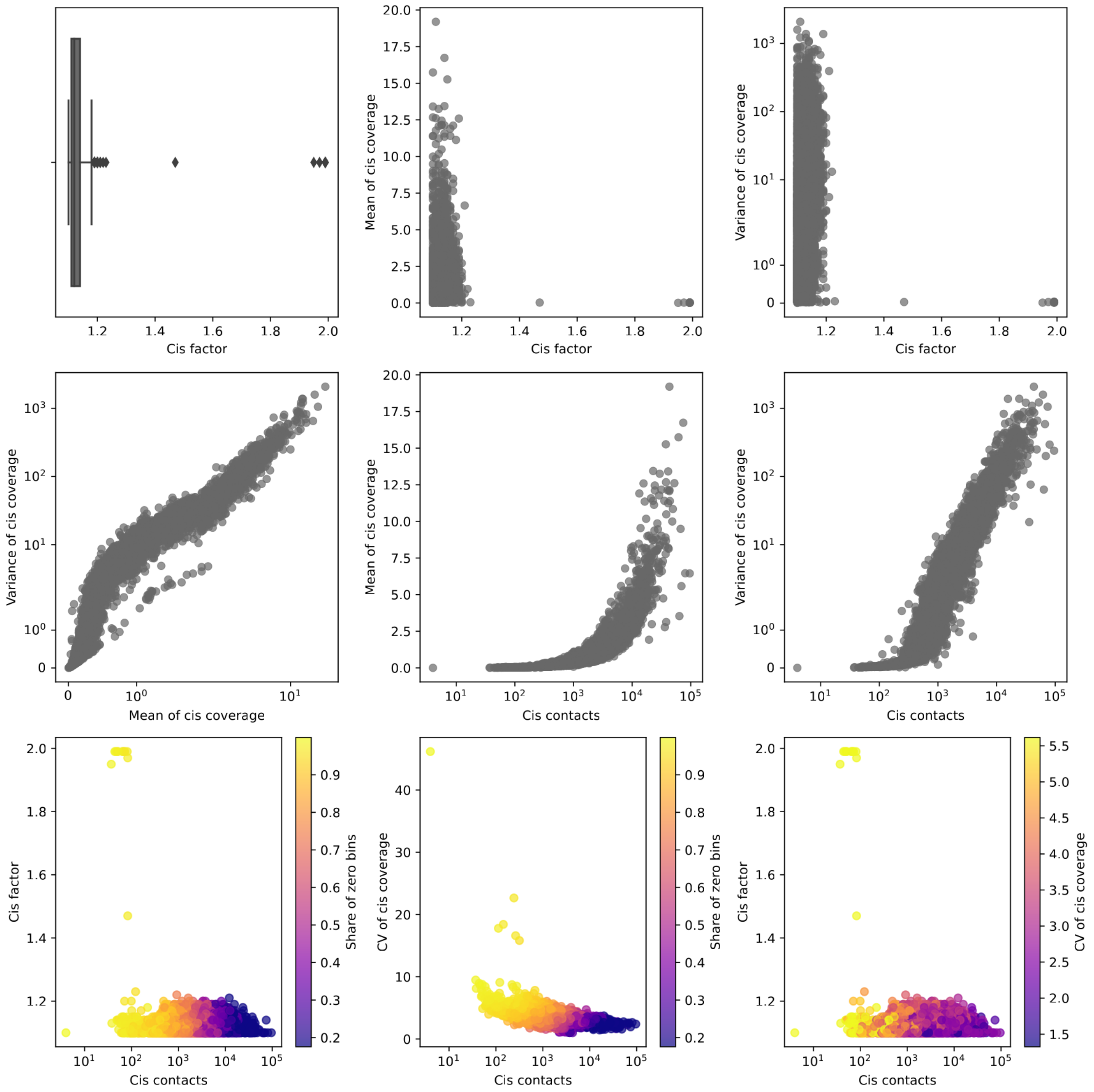

Supplementary Figure S1: Results of *cis factor* selection for RNAs from GRID data on mESC cell line. Only RNAs with more than 1000 contacts were considered. From left to right, from top to bottom: (1) *cis* factor distribution; the dependence of average *cis* bin coverage (2) and contact coverage variance (3) on bin size; 4) the ratio of average *cis* bin coverage by contacts to variance; the dependence of average *cis* bin coverage (5) and coverage variance (6) on the number of *cis* contacts; 7) the dependence of *cis* bins size on the number of *cis* contacts with a fraction of zero bins encoded by colour; 8) dependence of the coefficient of variation (CV) of *cis* bins coverage on the number of *cis* contacts, a fraction of zero bins encoded by colour; 9) the dependence of *cis* bin size on the number of *cis* contacts, CV of coverage encoded by colour.

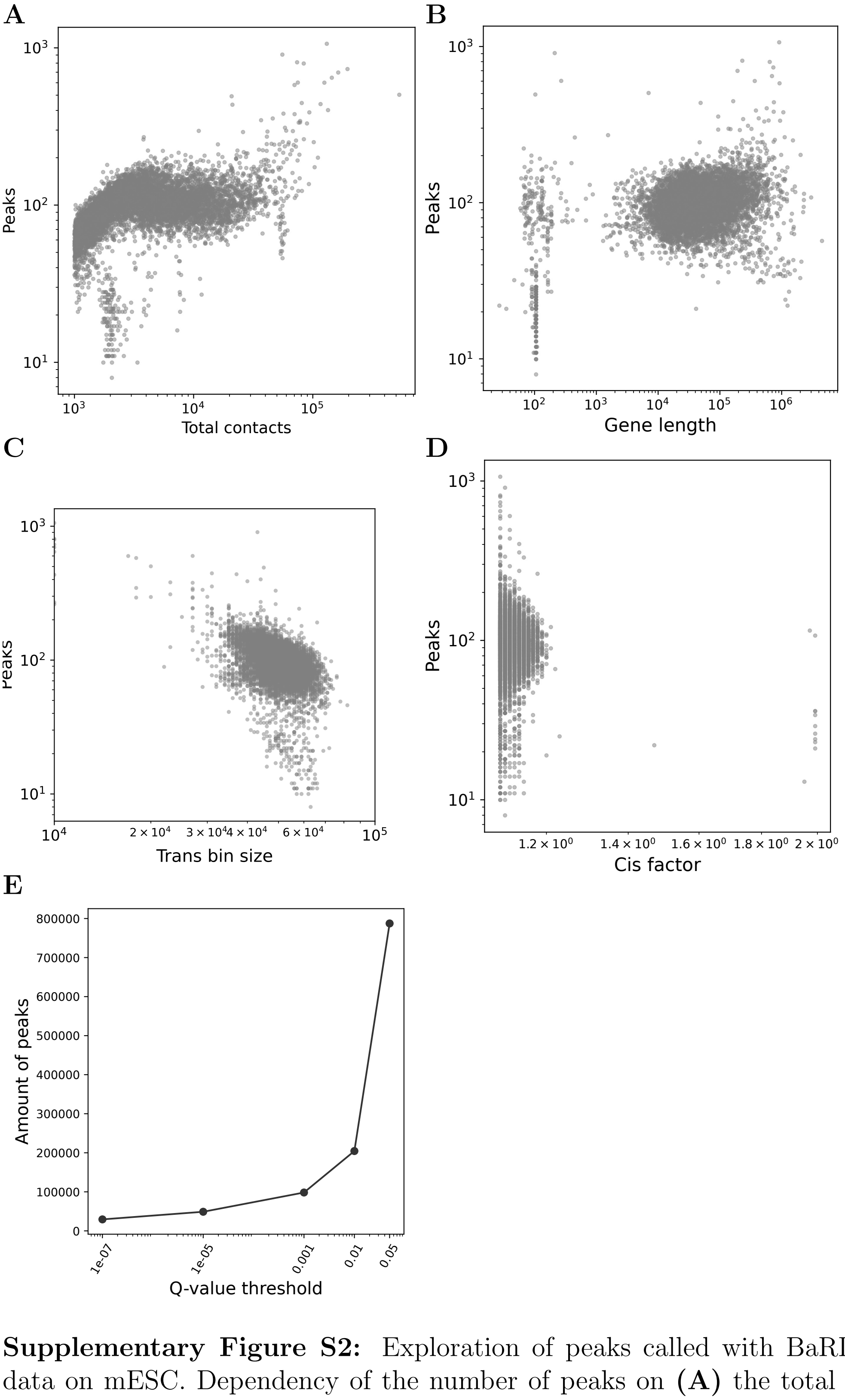

Supplementary Figure S2: Exploration of peaks called with BaRDIC from GRID data on mESC. Dependency of the number of peaks on **(A)** the total number of RNA contacts ($r_S = 0.45$), **(B)** the length of RNA source gene ($r_S = 0.23$), **(C)** selected *trans* bin size ($r_S = -0.44$), and **(D)** *cis* factor ($r_S = -0.02$) are shown. **(E)** Numbers of peaks at different Q-value thresholds, including mRNAs peaks.

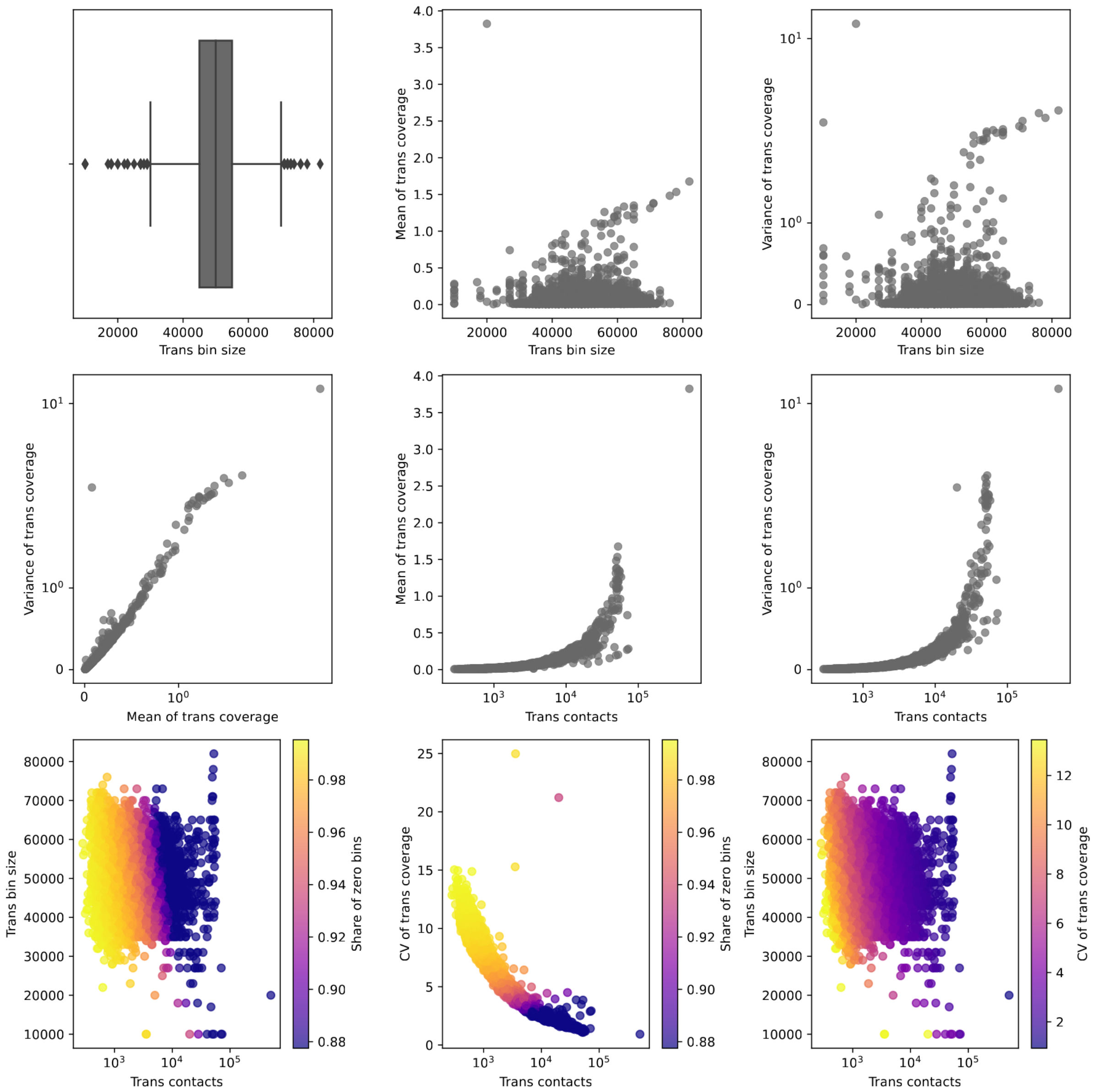

Supplementary Figure S3: Results of *trans bin* size selection for RNAs from GRID data on mESC. Only RNAs with more than 1000 contacts were considered. From left to right, from top to bottom: (1) *trans* bin size distribution; the dependence of average *trans* bin coverage (2) and contact coverage variance (3) on bin size; 4) the ratio of average *trans* bin coverage by contacts to variance; the dependence of average *trans* bin coverage (5) and coverage variance (6) on the number of *trans* contacts; 7) the dependence of *trans* bins size on the number of *trans* contacts with a fraction of zero bins encoded by colour; 8) dependence of the coefficient of variation (CV) of *trans* bins coverage on the number of *trans* contacts, a fraction of zero bins encoded by colour; 9) the dependence of *trans* bin size on the number of *trans* contacts, CV of coverage encoded by color.

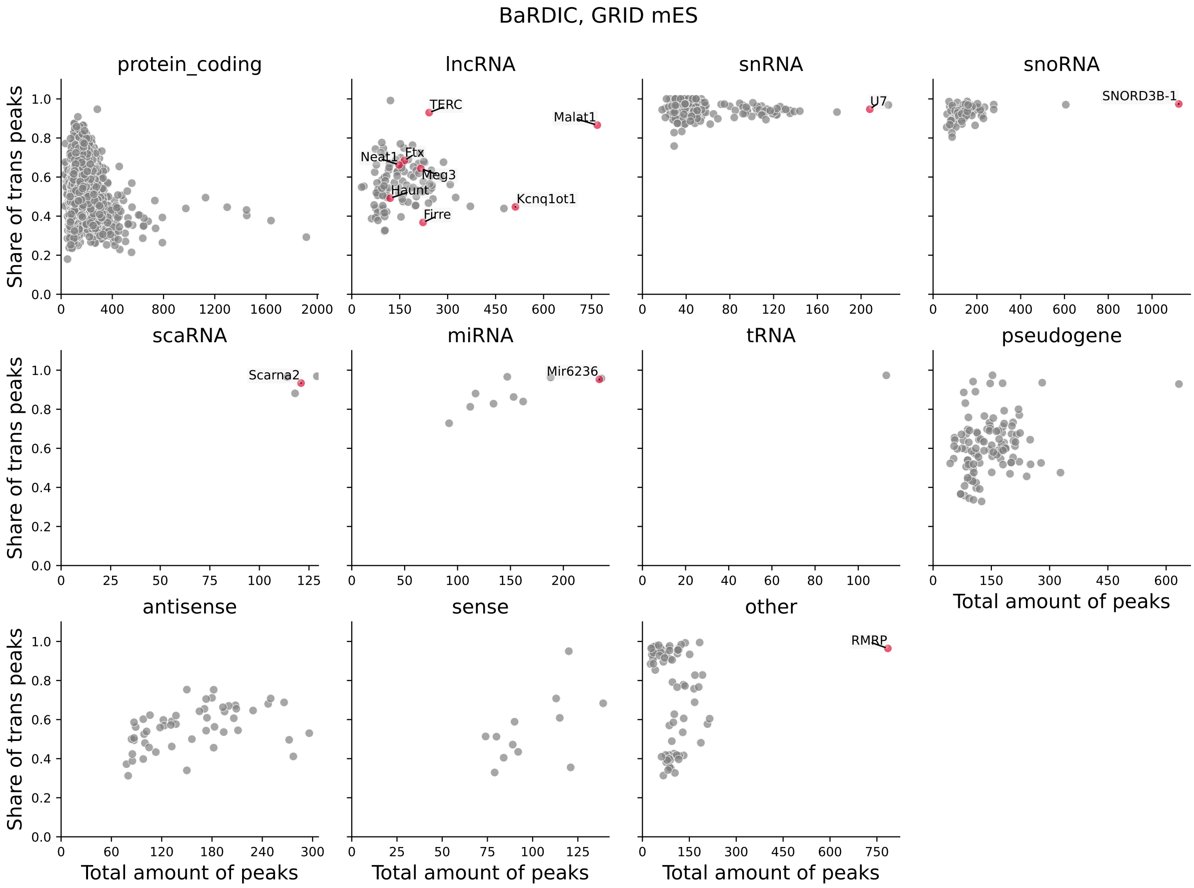

Supplementary Figure S4: Global assessment of specific RNA interactions identified by BaRDIC for GRID data on mESC data. Fractions of *trans* peaks and the number of ATA peaks for different RNA biotypes.

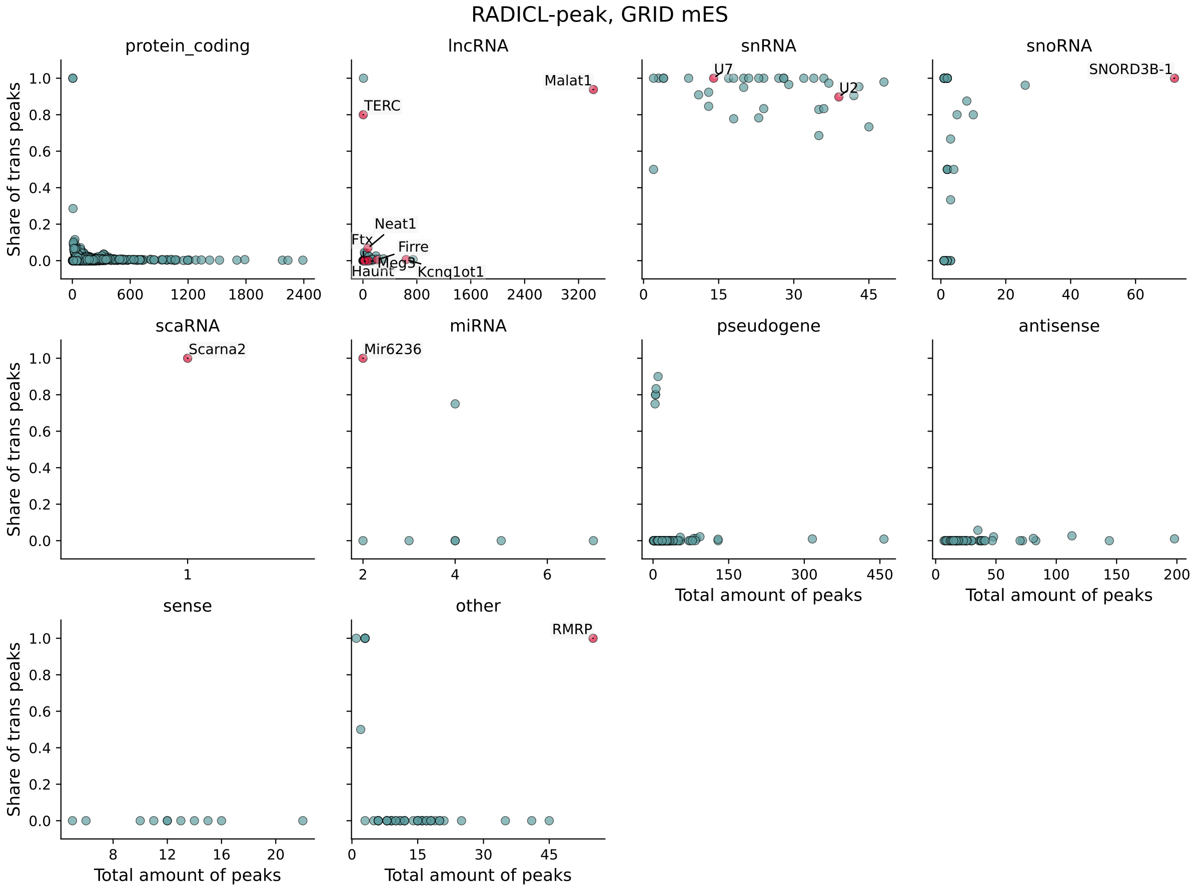

Supplementary Figure S5: Global assessment of specific RNA interactions identified by RADICL-peak for GRID data on mESC. Fractions of *trans* peaks and the number of ATA peaks for different RNA biotypes are shown. RADICL-peaks are called according to the algorithm described in the original study.

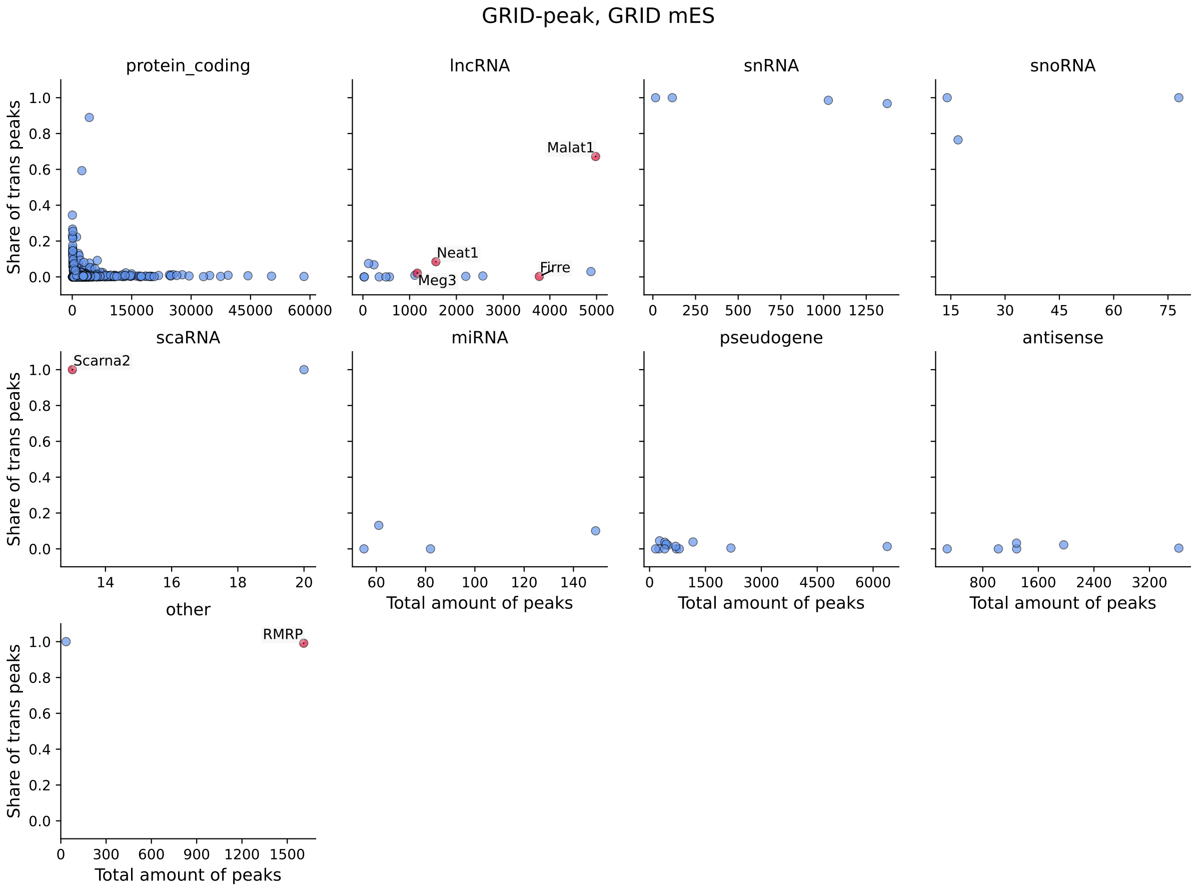

Supplementary Figure S6: Global assessment of specific RNA interactions identified by GRID-peak for GRID data on mESC. Fractions of *trans* peaks and the number of ATA peaks for different RNA biotypes are shown. Peak coordinates have been obtained from the GRID original study and converted into mm10 as described in the Materials and Methods.

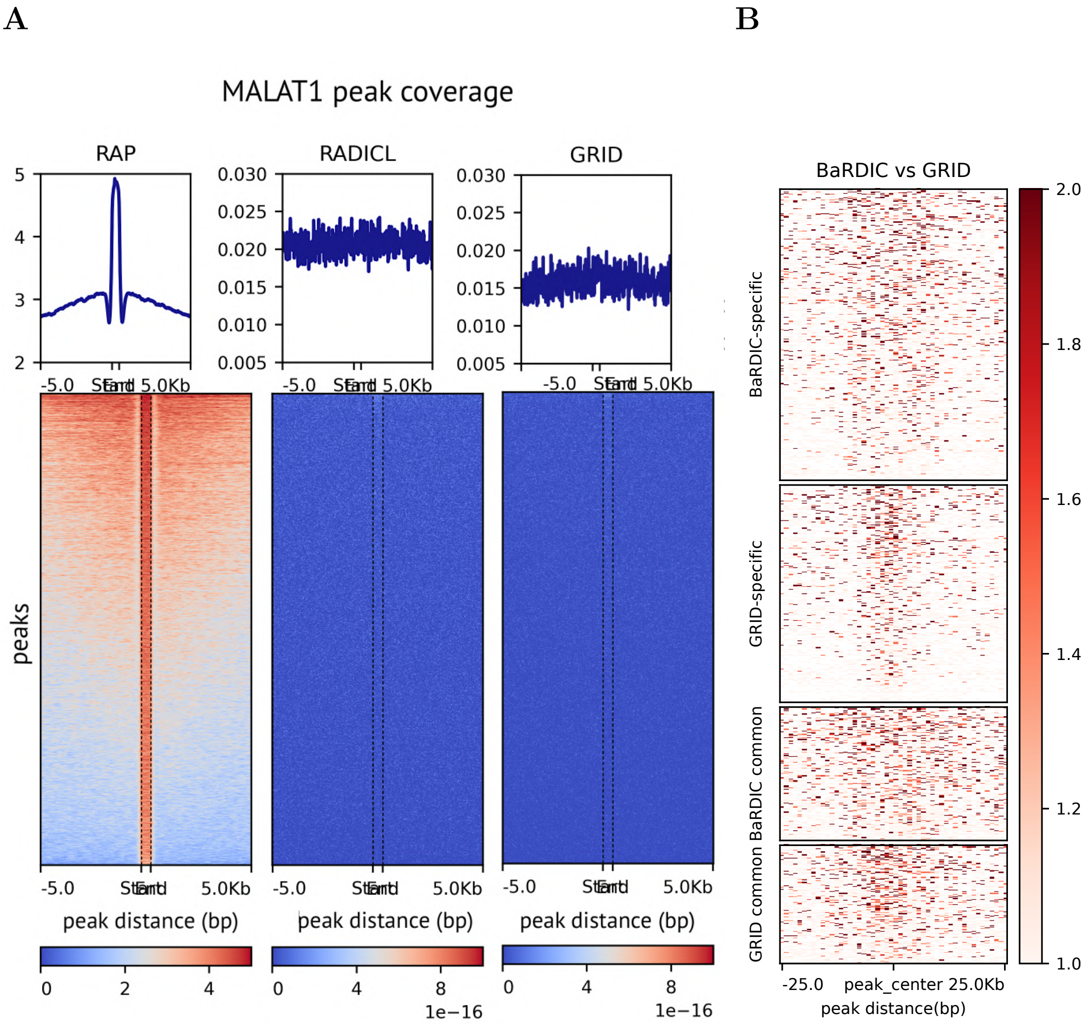

Supplementary Figure S7: (A) MACS2 peaks for Malat1 RAP OTA data and contacts from RAP, RADICL for GRID data, mESC. Coverage of OTA peaks with ATA contacts for mESC. **(B)** Coverage of common and algorithm-specific *trans* peaks identified by BaRDIC and GRID-peak with ATA contacts from GRID data on mESC cell line. Adjacent GRID-peaks were clustered as described in Supplementary Data Figure S13. Common peaks were defined as ones overlapping the same genes, and algorithm-specific as the others. From top to bottom: the contact coverage of BaRDIC-specific peaks, GRID-specific peaks, BaRDIC and GRID-peaks overlapping "common" genes.

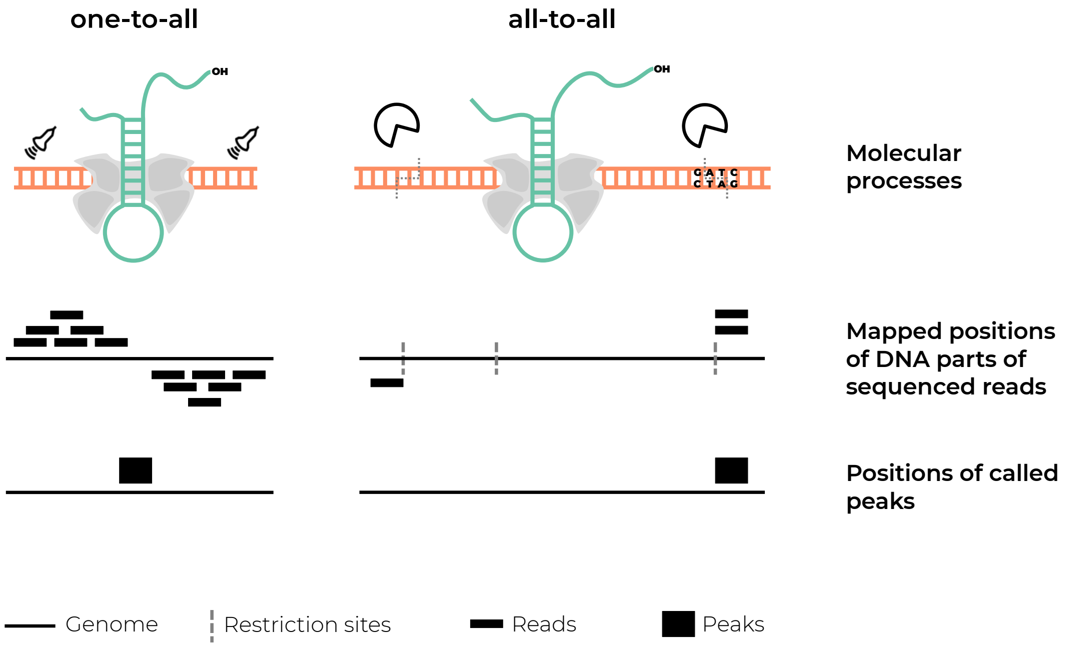

Supplementary Figure S8: For OTA experiments with DNA shearing, read form groups on both strands around the binding site. For all-to-all experiments with DNA digestion, DNA parts of contacts map to restriction sites downstream in 5' direction.

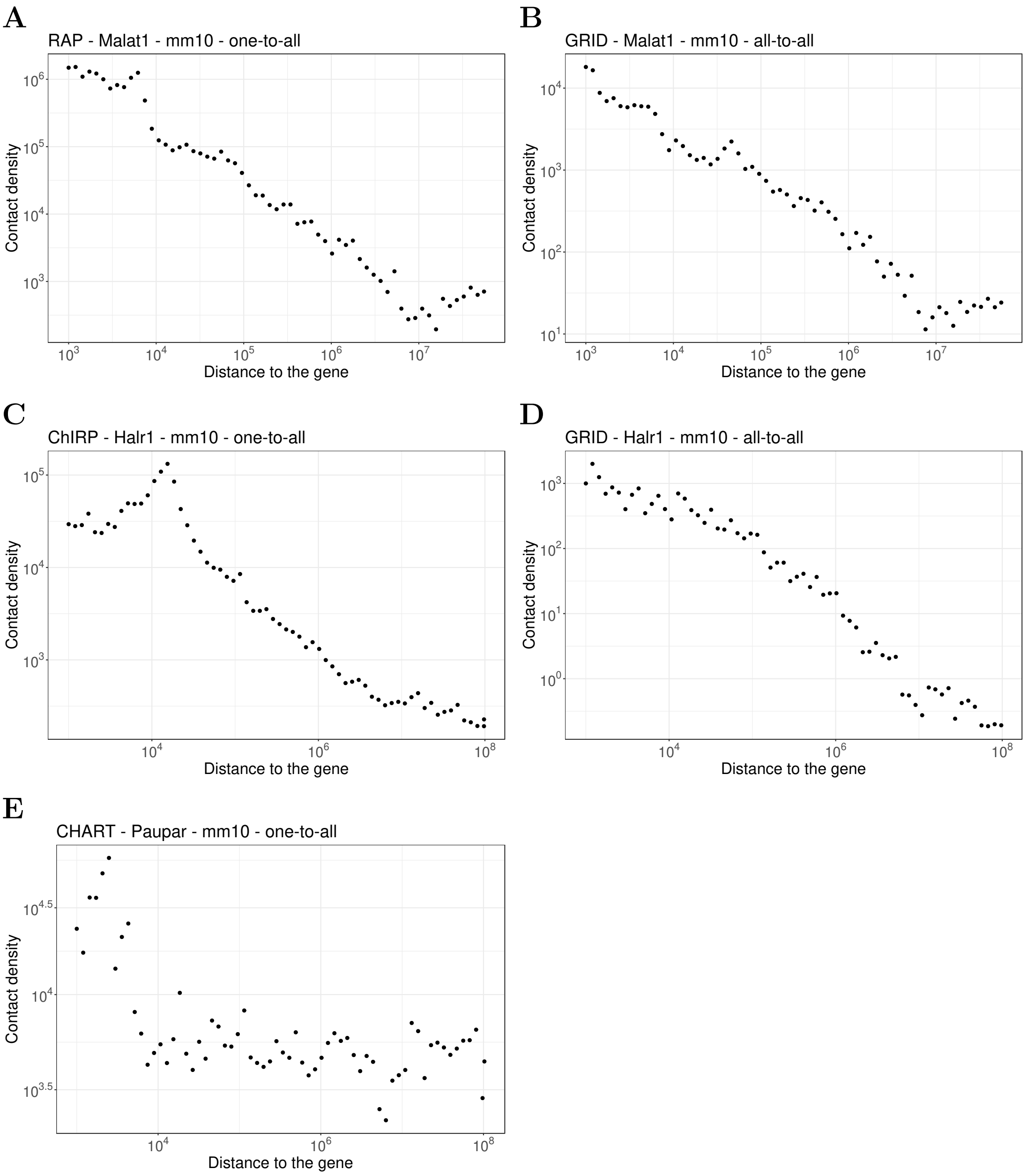

Supplementary Figure S9: Dependency of contact density on the distance between the RNA source gene and chromatin target loci (scaling) in double logarithmic coordinates. **(A)** Scaling of Malat1 in OTA RAP data, mESC **(B)** Scaling of Malat1 in ATA GRID data, mESC **(C)** Scaling of Halr1 in OTA ChIRP data, mESC. **(D)** Scaling of Halr1 in ATA GRID data, mESC. **(E)** Scaling of Paupar in OTA CHART data, N2A cell line.

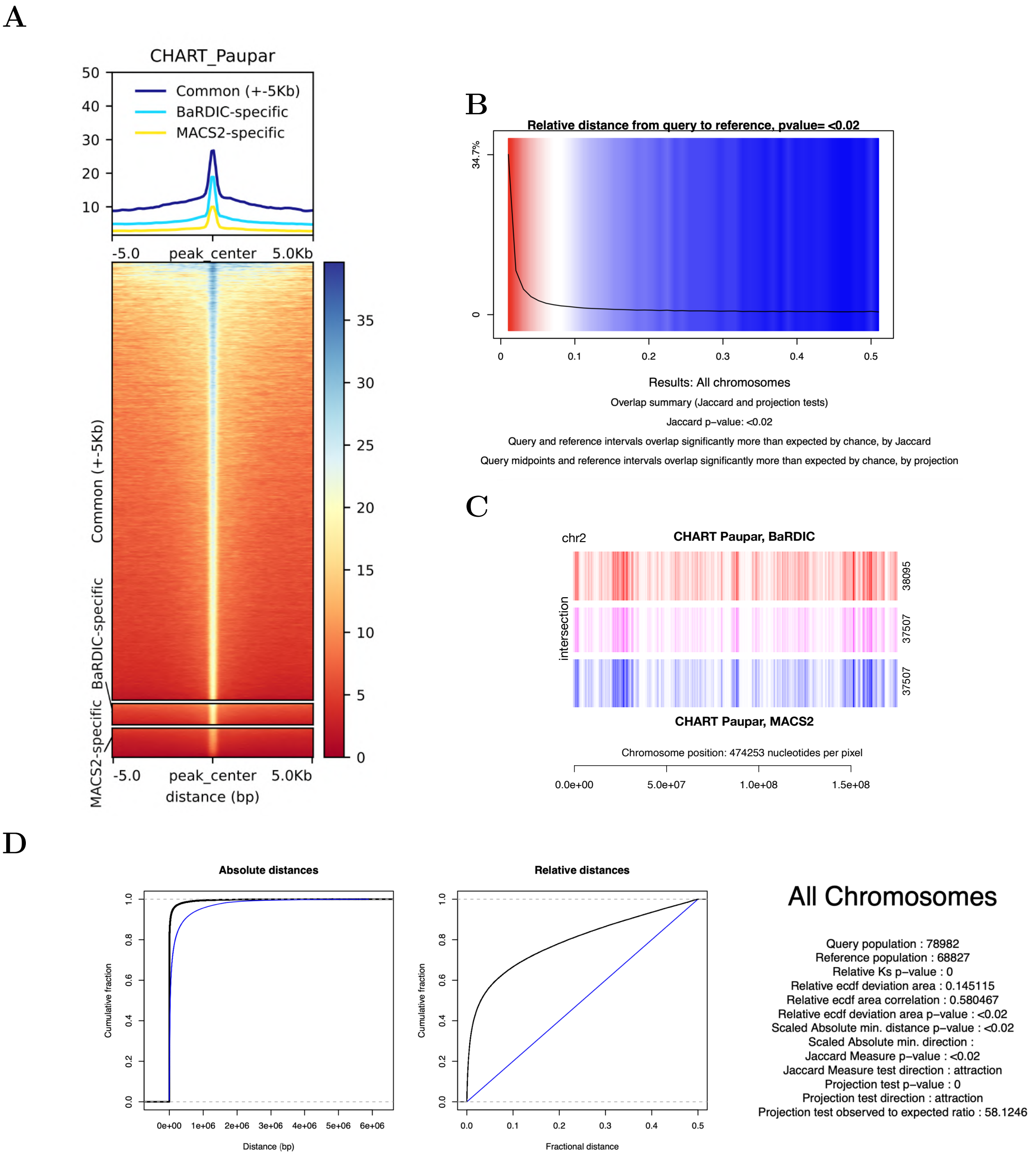

Supplementary Figure S10: Comparison of OTA peaks called with BaRDIC and MACS2 for CHART Paupar data, N2A cell line. **(A)** Coverage of OTA peaks with *trans* contacts. To identify non-reproducible peaks that are uniquely called by MACS2 or BaRDIC, we overlap peak sets (extended by 5Kb on each side) and assign them to categories: MACS2-specific, BaRDIC-specific or "common" peaks. For the latter, the profile was generated around BaRDIC peak centers. **(B)** GenometriCorr was used to examine pairwise spatial correlations between BaRDIC peaks (Query) and MACS2 peaks (Reference). The package implements statistical approaches that are based on interval overlaps and genomic distances. Areas of high and low relative distance correlation are shown: red and blue colours indicate deviation from the expected distribution, while the black line indicates the density of the data in these regions. **(C)** Graphical representation of BaRDIC and MACS2 peaks for chromosome 2 harboring source gene. **(D)** Statistical summary and ECDF plot for distance tests for the entire genome. The black line represents the actual distribution of data in comparison with expected distribution. Relative distance tests show a correlation, this indicates that BaRDIC and MACS2 peaks called for Paupar tend to co-localize.

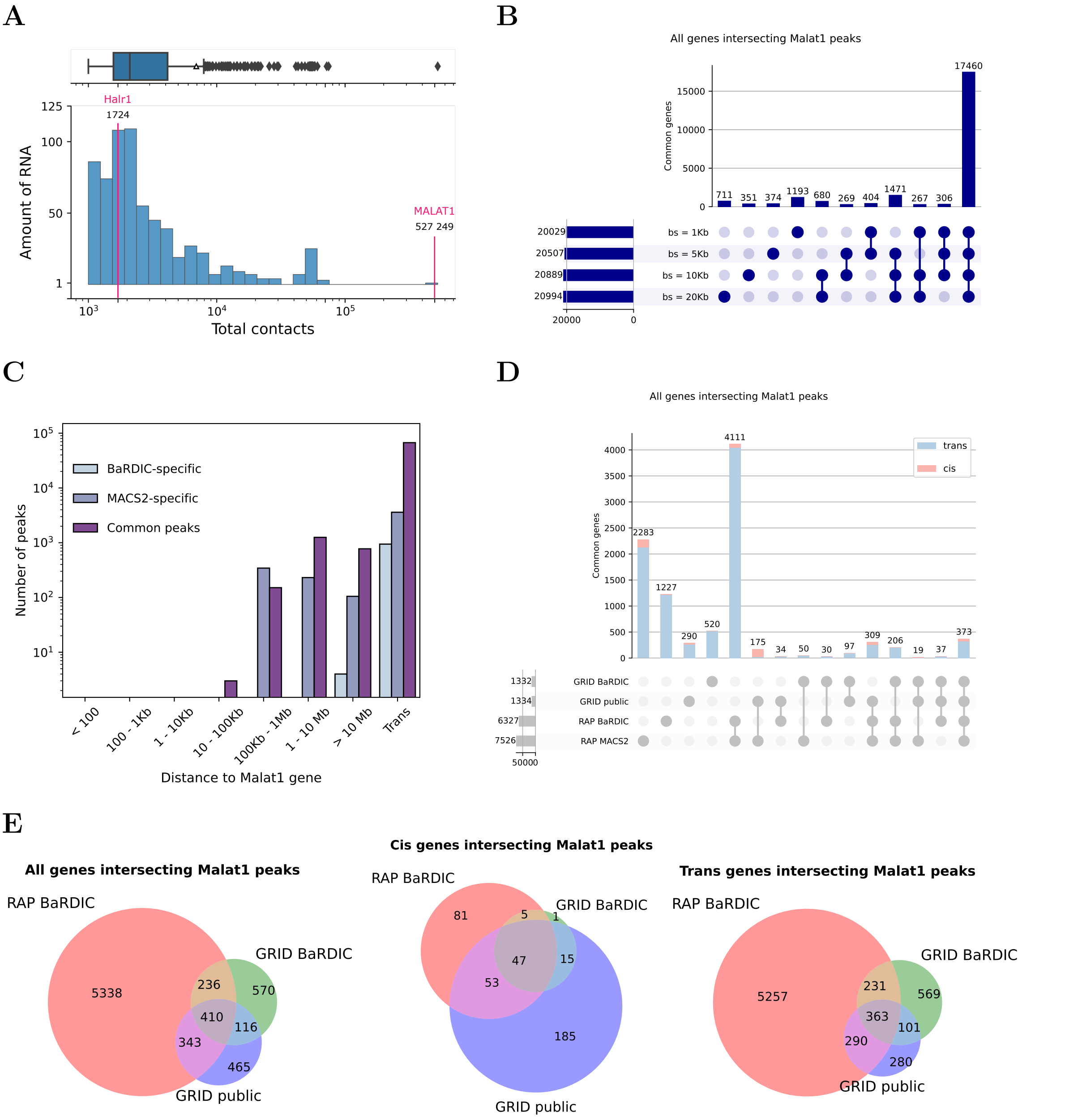

Supplementary Figure S11: **(A)** The distribution of numbers of contacts for all RNAs (> 1000 contacts) in GRID data on mESC except for rRNAs and mRNAs. Individual RNAs are shown: Malat1 as an example of an abundant RNA, and Halr1 as an example of an unabundant RNA. **(B)** BaRDIC peak calls with various bin sizes of the generated background track (1-20Kb). The UpSet plot shows the number of common genes overlapping Malat1 peaks in RAP data on mESC. **(C)** The number of peaks called with BaRDIC or MACS2 at a given distance from the Malat1 gene for RAP data on mESC. To identify non-reproducible peaks uniquely called by each method, we overlap peak sets (extended by 5Kb on both sides) and assign them to categories: BaRDIC-specific, MACS2-specific or "common" peaks. **(D)** The UpSet plot represents the numbers of *cis* and *trans* genes overlapping Malat1 peaks from RAP and GRID mESC data called with BaRDIC, MACS2 and GRID-peak. **(E)** Genes intersecting Malat1 peaks from RAP and GRID mESC data. From left to right: all genes, *cis* genes, *trans* genes.

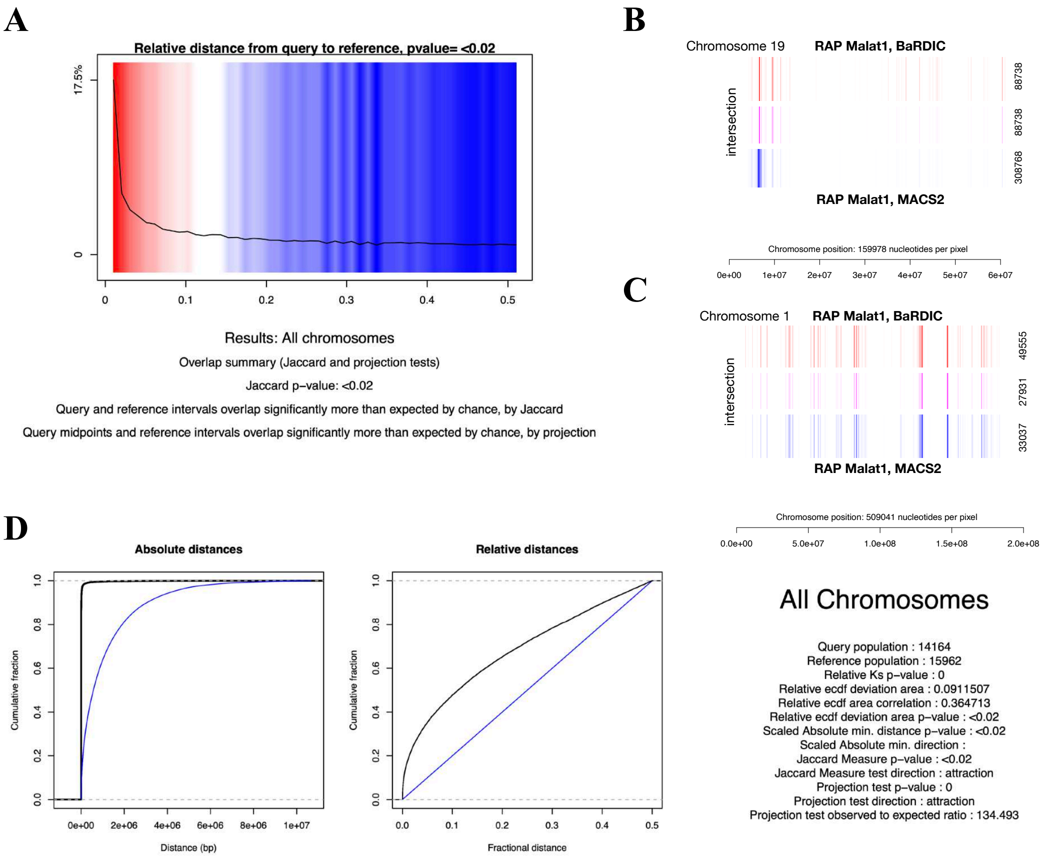

Supplementary Figure S12: GenometriCorr was used to examine pairwise spatial correlations between genomic profiles: BaRDIC peaks (Query) and MACS2 peaks (Reference) called from RAP Malat1 data. **(A)** Areas of high and low relative distance correlation. Red and blue colours indicate deviation from the expected distribution, while the black line indicates the density of the data in these regions. **(B)** Graphical representation of genomic profiles for chromosome 19 harbouring Malat1 source gene. **(C)** Graphical representation of BaRDIC and MACS2 peaks for "non-parental" chromosome 1 **(D)** Statistical summary and ECDF plot for the relative and absolute distances for the entire genome. The blue line represents the expected distribution (no association), and the black line represents the actual distribution of data. Relative distance tests show a correlation, this indicates that BaRDIC and MACS2 peaks for Malat1 tend to co-localize.

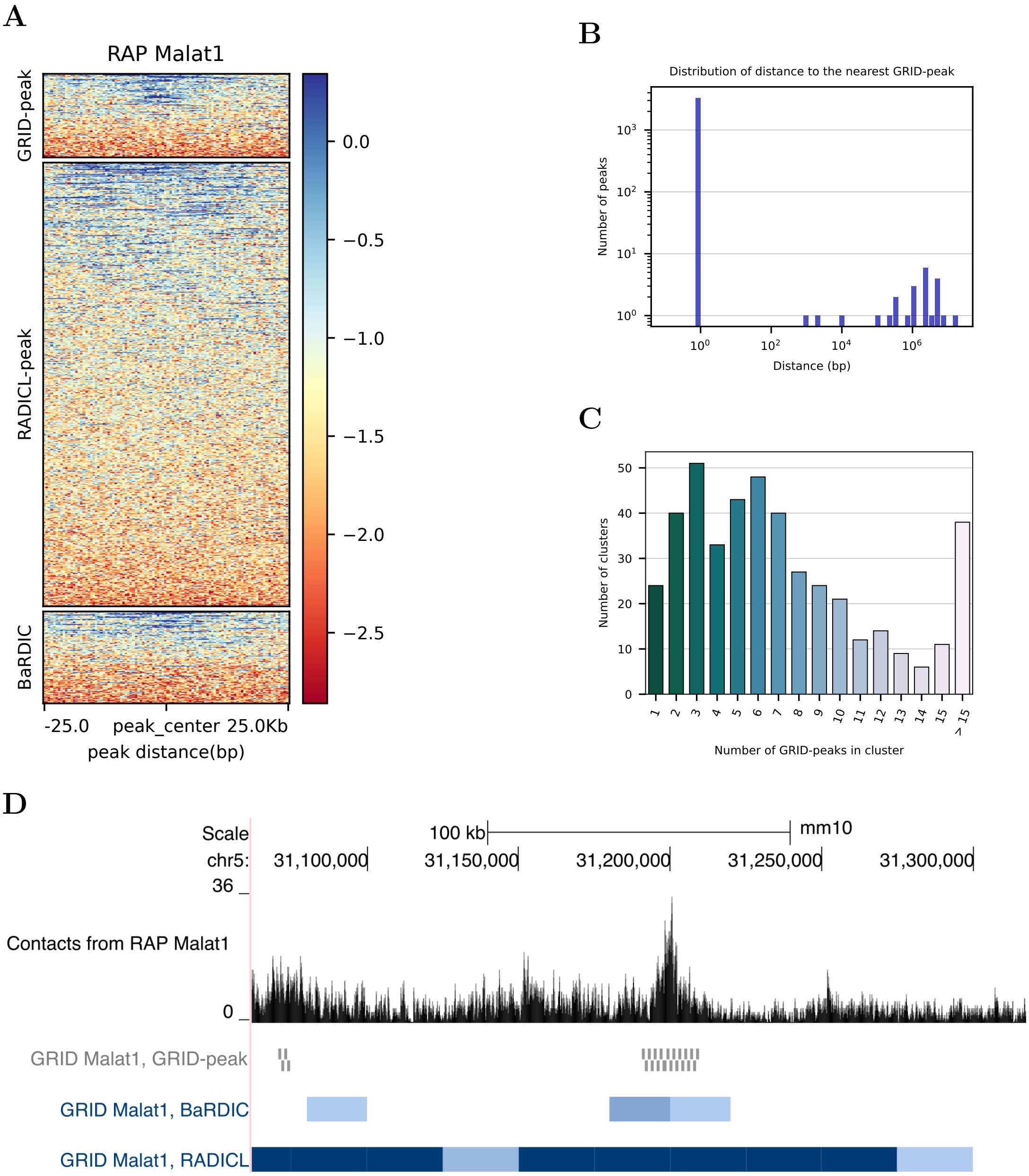

Supplementary Figure S13: Comparison of ATA Malat1 peaks from the GRID experiment with OTA RAP contacts on mESC. **(A)** Coverage of ATA peaks for Malat1 obtained by three algorithms (BaRDIC, RADICL-peak, GRID-peak) with RAP *trans* contacts. The RAP signal trace is normalised to the background. As GRID peaks are relatively narrow (1Kb) and are co-localised, we merged adjacent peaks into larger regions. **(B)** Distribution of distances to the nearest peak for GRID-peak peaks obtained from the original study. **(C)** Distribution of numbers of GRID-peak peaks in each merged peak region. **(D)** Genomic browser view of peaks called with three algorithms in comparison with OTA Malat1 contacts.

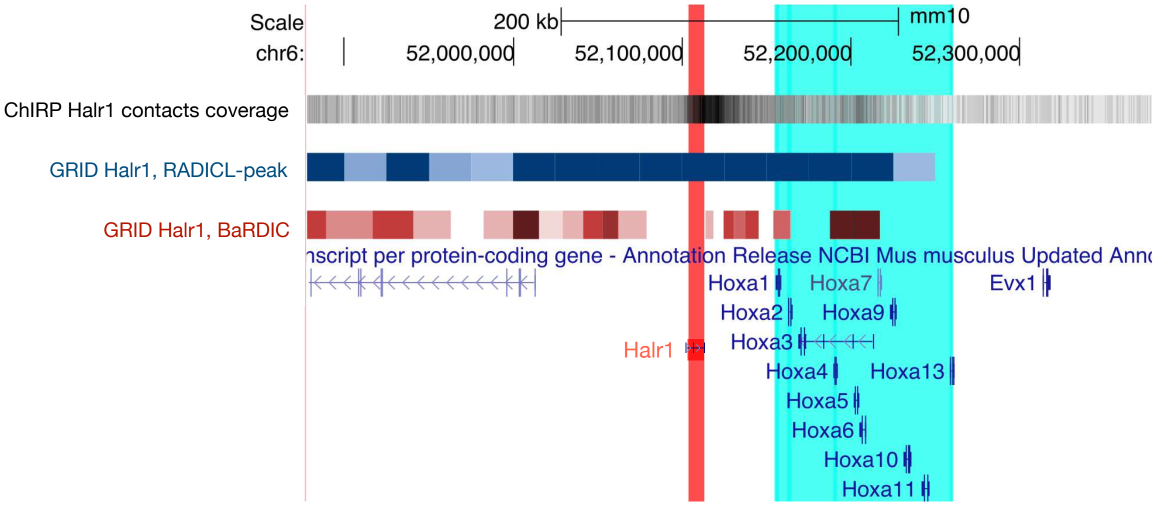

Supplementary Figure S14: Representative genome browser view of Halr1 peaks called with BaRDIC and RADICL-peak for GRID ATA data on mESC. Halr1 source gene (red) and HoxA gene cluster (cyan) are highlighted. RADICL peaks are 25 Kb wide, while BaRDIC initial *cis* bin size equals 5 Kb with *cis* factor = 1.12.
