# Supplementary material for: BaRDIC: robust peak calling for RNA–DNA interaction data
Source: NAR Genom Bioinform. 2024 May 20;6(2):lqae054. doi: 10.1093/nargab/lqae054 (PMC11106031; doi:10.1093/nargab/lqae054)
Supplement: lqae054_Supplemental_Files [file lqae054_supplemental_files.zip › NARGAB_2023_163_R1_Suppl_Note_rev2.pdf]

Supplementary Notes

I. BaRDIC algorithm

The BaRDIC algorithm consists of three steps:

1. Binning: for each RNA, chromosomes are partitioned into non-overlapping genomic intervals (bins), and the number of contacts is calculated within bins.

2. Statistical modelling: for each RNA, parameters of the background model are calculated in every bin. P-values are calculated based on them.

3. Multiple testing correction with Benjamini-Hochberg procedure [1].

To increase the statistical power and speed up the algorithm's performance, by default, only RNAs with more than 1000 contacts are selected. Steps 1 and 2 are atomic with regard to individual RNAs, so we describe them for one RNA only.

1 Binning

Bin sizes are estimated for each RNA separately. The binning strategy differs for *cis* interactions, which occur on a chromosome harbouring the RNA gene, and *trans* interactions – with other chromosomes. To find optimal bin sizes, we adapt RSEG [2] and JAMM [3] approaches that were initially developed for ChIP-seq peak-calling. This approach finds a balance between resolution and sufficient bin coverage.

1.1 Binning in *trans*

In the case of random ligation, we expect that *trans* contacts are distributed uniformly along chromosomes. Therefore, we apply a uniform binning strategy

for *trans* interactions. Selection of the optimal bin size for *trans* interactions is done via minimizing the cost function C:

$$\hat{trans\ bin} = argmin\ C(binsize) = argmin \frac{2 \cdot mean - var}{binsize^2},$$

where *mean* is the average number of contacts in bins, *var* is the variance of the number of contacts in bins, and *binsize* is the corresponding *trans binsize* in nt.

1.2 Binning in *cis*

For binning in *cis* scaling must be taken into account. Also, we assume long-range *cis* interactions are similar to *trans* interactions, analogous to observations in Hi-C data analysis [4]. Since contact densities at different distances can differ by orders of magnitude, we abandoned the uniform binning: chromosomes are partitioned into non-uniform bins of size increasing in a geometric progression from source gene boundaries. *Cis* bin size increases until it exceeds the *trans* bin size; all subsequent *cis* bins are uniform and equal to the *trans* bin in size. This strategy achieves:

1. Uniform coverage of bins by contacts, which is required by the bin size optimization procedure.
2. Higher resolution near the source gene and more accurate estimation of *cis* peaks.
3. Similarity of distant *cis* and *trans* bins.

Finally, the size of a *cis* bin with sequence number i from the gene boundaries of a particular RNA is calculated using the formula:

$$cis\ binsize(i) = \min(startsize \cdot factor^i, trans\ binsize),$$

where *startsize* is a tunable user-specified parameter, while $factor$ is optimized by minimizing the cost function similarly to the *trans* bin size selection:

$$C(factor) = \frac{2 \cdot mean - var}{factor^2}.$$

1.3 Cost function minimization

We found that in our data, the cost function may or may not have a global minimum (Note Figure 1A, B), so standard function minimization algorithms cannot be applied. Instead, we developed a problem-tailored minimization approach.

We want to find such a bin size that it is not too small (otherwise each bin will have too few contacts, which will reduce statistical power) and not too large (otherwise resolution will be too low). So we start with some small bin size (*start binsize*), increase it by the same value (*step*) and for each bin size

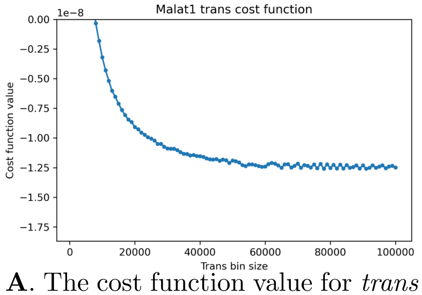

A. The cost function value for *trans* bins

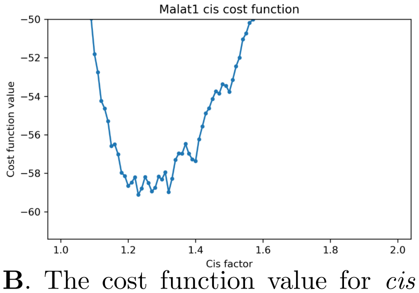

B. The cost function value for *cis* factor

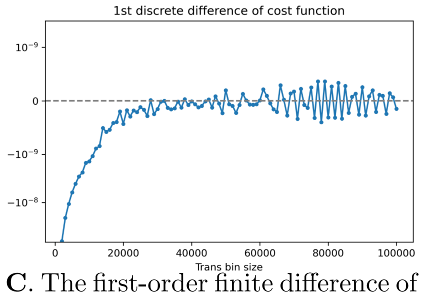

C. The first-order finite difference of the cost function for *trans* bins

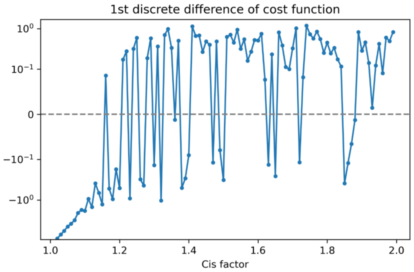

D. The first-order finite difference of the cost function for *cis* factor

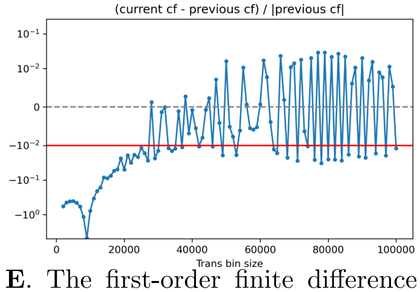

E. The first-order finite difference divided by the cost function absolute value for *trans* bins.

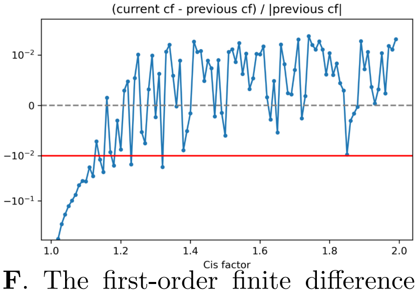

F. The first-order finite difference divided by the cost function absolute value for *cis* factor.

Note Figure 1: The optimization process on *trans bin* size and *cis factor* values for MALAT1, GRID-seq data on mESC.

$\{start\ binsize + k \cdot step\}_{k=0}^{\infty}$ we calculate the cost function $\{C_k\}_{k=0}^{\infty}$. Consider the first-order finite difference of the cost function: $\Delta C_k = C_{k+1} - C_k$. Note that it tends to zero as the bin size increases, and then starts to oscillate around it (Note Figure 1C, D). This behaviour means that the rate of decrease of the cost function decreases with increasing bin size up to some point, and then the cost function goes up and down. So, we want to find a point at which the cost function:

1. Starts decreasing too slowly (there is no global minimum);
2. Or it starts to grow (there is a global minimum).

The second case is defined as $\Delta C_k > 0$, and for the first case, we have to introduce some threshold $\delta > 0$ and find a bin size such that $\Delta C_k < \delta$. Since the scale of the cost function will be different for different RNAs and for *trans* and *cis* bins as well, we divide the finite difference by the value of the cost function and take the absolute value of the resulting relative finite difference: $f_k = \frac{\Delta C_k}{|\Delta C_k|}$ (Note Figure 1 E, F). Then the point of the minimum of the cost function is such a bin size $start\ binsize + kstep$, that corresponds to the smallest k at which one of the three conditions is satisfied:

1. $\Delta C_k > 0$ (the cost function grows);
2. $f_k < \delta$ (the cost function converges);
3. $start\ binsize + k \cdot step > max\ binsize$ (limit, the cost function doesn't converge).

The physical meaning of this optimization procedure is as follows: we choose the smallest bin size that for the (next) larger bin size the cost function either grows or decreases slower than $\delta \cdot 100\%$, or we choose the largest bin size allowed ($max\ binsize$).

For some RNAs, the cost function fluctuates too much for our minimization procedure to work correctly. So the cost function can be smoothed over consecutive bin sizes by taking the average value of the cost function over the previous w steps:

$$C_k^{smooth} = \frac{1}{w} \cdot \sum_{h=k-w+1}^{k} C_h.$$

By default, no smoothing is performed, i.e. $w = 1$.

The parameters for the optimization procedure were chosen with respect to the resolution of ATA experiments and are presented in Note Table 1.

**Parameter**	***Trans bin***	***Cis factor***
start bin size	10 Kb	1,1
step	1 Kb	0,01
max bin size	1 Mb	2
δ	0,01	0,01

Note Table 1: Empirically selected parameters for *cis* and *trans* bins size optimization for all-to-all RNA-DNA interaction data. Set as default parameters of BaRDIC algorithm.

2 Statistical modeling

2.1 Background model

To model the background distribution of RNA-DNA contacts in bins, we introduce a frequentist model similar to those used in ChIP-seq and Hi-C data analysis [5, 6]. Assuming that contacts arising from random binding are independent, we consider the number of contacts X_{ij} of RNA i in bin j to be binomially distributed:

$$X_{ij} \sim Bin(N_i, p_{ij}),$$

where N_i is the total number of contacts produced by RNA i (except the gene body), p_{ij} is the background probability. Note the number of observed contacts O_{ij} is a realization of the random variable X_{ij}. Statistical modelling comes down to inferring the only model parameter p_{ij} from the observed data.

2.2 Inference for *trans* bins

For *trans* bins, only chromatin heterogeneity plays a role. For ATA experiments, we estimate the parameter of the background model by counting mRNA *trans* contacts as proposed in the GRID-peak procedure. The background probability of a single contact of RNA i to appear in the j-th *trans* bin is defined as follows:

$$\hat{p} = \hat{p}_j^{bg} = \frac{N_j^{bg}}{N^{bg}},$$

where N_j^{bg} is the number of contacts from the background in a bin j, and N^{bg} is the total number of background contacts. For OTA experiments, we use contacts from the input sample similar to ChIP-seq analysis.

2.3 Inference for *cis* bins

To estimate the background probability for *cis* bins, we have to additionally consider scaling. Inspired by Hi-C analysis methods [5], we define the corresponding probability as follows:

$$\hat{p_{ij}} = f(d_{ij}) \cdot \hat{p_j^{bg}},$$

where d_{ij} is the distance between the midpoint of bin j and the closest boundary of RNA i source gene, $f(d_{ij})$ – is the scaling factor. Assuming $O_{ij} \approx EX_{ij} = N_i\hat{p_{ij}}$, we can estimate $f(d_{ij})$ as follows: $\hat{f}(d_{ij}) = \frac{O_{ij}}{N_i \cdot \hat{p_j^{bg}}}$.

Since the bins on the 5'- and 3'-sides of a source gene are at the same distance from the gene, there are two values of $\hat{f}$ for each d_{ij}; however, there should be only one value of f. We calculate f using a smoothing B-spline of degree 3 in double logarithmic coordinates. It turns out that for individual RNAs from a single chromosome, $\hat{f}(d_{ij})$ may differ by 2-3 orders of magnitude in (Note Figure 2), probably due to the sparsity of the data. Accordingly, the spline is calculated separately for each RNA.

To exclude potential *cis* peaks from the background model, we estimate scaling factors in 2 steps similar to Fit-Hi-C [5] and HiC-DC [7]. In the first step, we compute spline-1 over all bins j for each RNA i, then determine p-values by right-sided binomial test for each *cis* bin and remove those bins with $pvalue = \frac{1}{M_i^{cis}}$, where M_i^{cis} is the number of non-zero *cis* bins for RNA i. If $\frac{1}{M_i^{cis}}$ is greater than the threshold value 0.05, the latter is used. This filtering excludes likely specific binding sites. We then compute spline-2 in the same manner using only the remaining bins. When computing both splines, we remove all bins j for which $O_{ij} = 0$ and/or $N_j^{bg} = 0$ to avoid spline distortion due to data sparsity.

The final scaling factor for each RNA i and each bin j is calculated as the geometric mean of three scaling factors: spline-2 values for the beginning of the bin, the middle of the bin, and the end of the bin. "1" is added to each relative coordinate to avoid zeros when taking logarithms.

2.4 Treating zero values

In both *cis* and *trans* inference, except spline fitting, the background coverage value $N_j^{bg} = 0$ is treated as missing and imputed with a very small positive value. We define it as the average base-pair coverage of the genome by background contacts, multiplied by the length of bin j and by a reducing imputation factor (0.01 by default).

2.5 Parameter renormalization

To hold $\sum_j \hat{p}_{ij} = 1$, we renormalize probability estimates for each RNA i. For this purpose, it is easiest to partition the probability space of the whole genome into two subspaces: the source RNA chromosome and the other chromosomes, so that the sum of the background probabilities for *cis* bins equals the fraction of *cis* contacts of this RNA (similarly for *trans* bins). For this purpose, let us introduce some more notations:

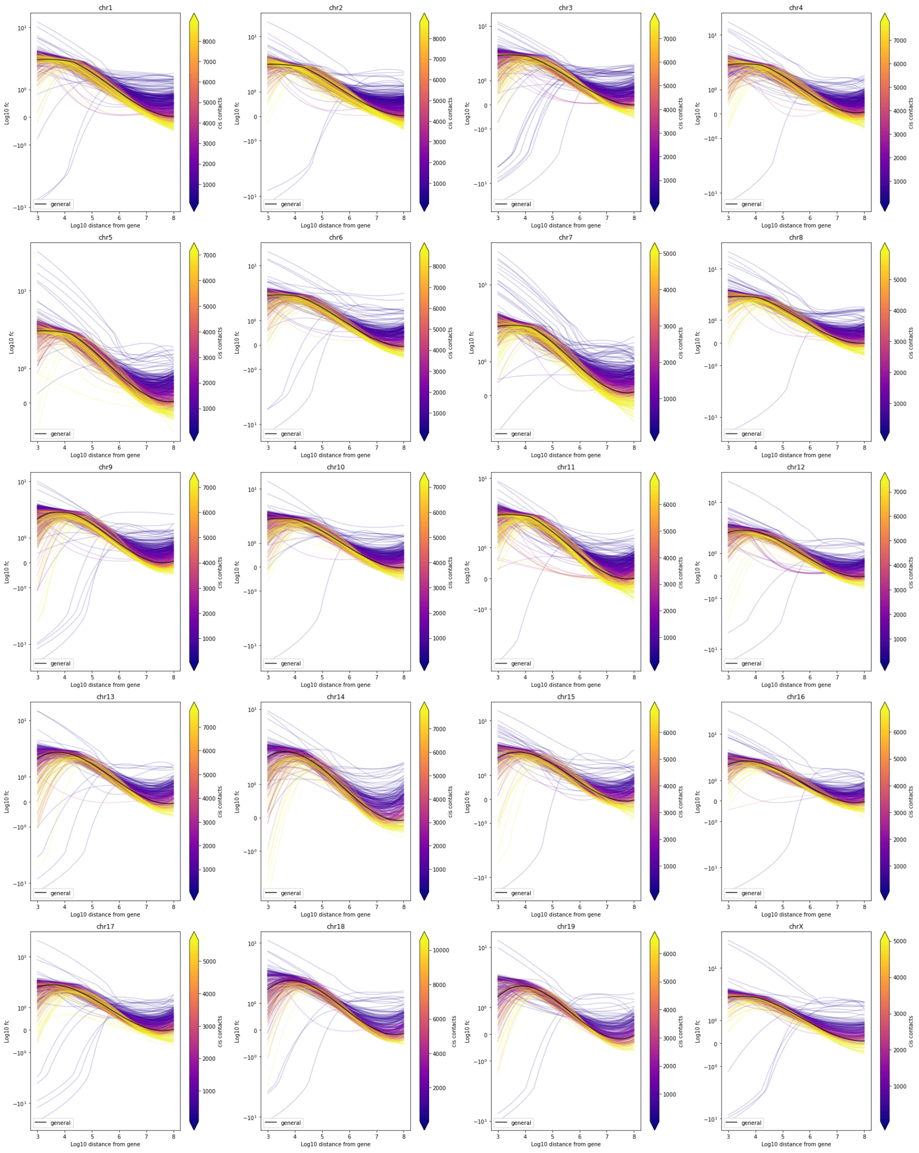

Note Figure 2: Scaling splines of individual RNAs in all-to-all GRID data on mESC cell line.

**Notation**	**Meaning**
$C(i)$	The source chromosome of RNA i
$C(j)$	The chromosome of bin j
$N_i^{cis} \equiv \sum_{j:C(i)=C(j)} O_{ij}$	The number of *cis* contacts of RNA i
$\hat{p}_j^{bg^{cis}} \equiv \frac{N_j^{bg}}{\sum_{j:C(i)=C(j)} N_j^{bg}}$	The frequency of background contacts in *cis* for RNA i
$\hat{p}_j^{bg^{trans}} \equiv \frac{N_j^{bg}}{\sum_{j:C(i)\neq C(j)} N_j^{bg}}$	The frequency of background contacts in *trans* for RNA i

Then, renormalized estimated background probabilities $\hat{p}_{ij}$ are calculated as follows:

**Type of bin**	**Background probability**	**Sum of background probabilities**
trans	$\hat{p}_{ij}^{trans} = \hat{p}_j^{bg^{trans}} \cdot \frac{N_i - N_i^{cis}}{N_i}$	$\sum_j \hat{p}_{ij}^{trans} = \frac{N_i - N_i^{cis}}{N_i}$
cis	$\hat{p}_{ij}^{cis} = \frac{\hat{f}_i(d_{ij}) \cdot \hat{p}^{bg^{cis}}}{\sum_{j:C(i)=C(j)} \cdot \hat{f}_i(d_{ij}) \cdot \hat{p}^{bg^{cis}}} \cdot \frac{N_i^{cis}}{N_i}$	$\sum_j \hat{p}_{ij}^{cis} = \frac{N_i^{cis}}{N_i}$

These background probabilities are the final parameter values ($\hat{p}_{ij}$) of the background binomial model.

2.6 P-value calculation

We expect that a specific binding event results in a high enrichment of contacts relative to the background model. The resulting $p - value$ is then computed with the right-sided binomial test using estimated background parameters:

$$pvalue(O_{ij}|X_{ij}) = P_{Bin}(X_{ij} \geq O_{ij}|N_i, \hat{p}_{ij}) = \sum_{k=O_{ij}}^{N_i} \binom{N_i}{k} \cdot \hat{p}_{ij}^k \cdot (1 - \hat{p}_{ij})^{N_i - k}$$

3 Multiple testing correction

P-values from non-zero bins are subjected to multiple testing correction using the Benjamini-Hochberg procedure, simultaneously for all RNAs. This procedure controls FDR on the global level, for all peaks of all RNAs in an ATA dataset. So the same FDR level cannot be propagated to peaks of individual RNAs based on this procedure.

4 Implementation

4.1 Software

The algorithm is implemented as a package for Python 3 [8] with a command line interface and is available at https://github.com/dmitrymyl/BaRDIC. It uses several packages for scientific computing (see the table below). Since binning and statistical evaluation are performed for each RNA separately, these steps are parallelized.

Package name	Version	Usage	Reference
numpy	1.24.4	Vector data operations	[9]
pandas	2.0.3	Table data operations	[10]
scipy	1.10.1	Splines and binomial tests	[11]
statsmodels	0.14.0	FDR correction	[12]
bioframe	0.4.1	Operations on genomic intervals	[13]
h5py	3.9.0	Access to HDF5 storage	[14]
tqdm	4.65.0	Progress bars and process-based parallelization via concurrent.futures	[15]

4.2 Data storage

To organize the data storage and speed up the data access, we developed two HDF5-based data formats [14].

4.2.1 dnah5 file format

This file format is a binary representation of DNA parts of contacts grouped by individual RNAs with optimized bin sizes. The layout of the dnah5:

```
/
- chrom_sizes/
    - chrom | O
    - size | int64
- dna_parts/
    - rnaN/
        - chromN/
            - start | int64
            - end | int64
```

File-level attributes:

Attribute	Type	Description
are_binsizes_selected	bool	Whether bin sizes are selected for each RNA and corresponding data is recorded in the file
version	str	dnah5 schema version. Currently, only "1" is available

RNA-level attributes contain coordinates of the source gene, contact statistics, and binning parameters:

Field	Type	Description
chrom	O	RNA gene chromosome
start	int64	RNA gene start
end	int64	RNA gene end
total_contacts	int64	Total number of RNA contacts
genic_contacts	int64	Number of RNA contacts inside its gene
*cis*_contacts	int64	Number of *cis* RNA contacts: on the RNA's origin chromosome but outside RNA gene
*trans*_contacts	int64	Number of *trans* RNA contacts: on all chromosomes except for RNA's origin one
eligible	bool	Whether this RNA has enough contacts for further processing
*cis*_factor	float64	*cis* factor value for binning
*cis*_start	int64	Initial *cis* bin size
*trans*_bin_size	int64	*trans* bin size

4.2.2 rdc file format

This file format holds binned RNA-DNA contacts and corresponding values as well as the binned background track. Bin tables are organized for each RNA separately. The layout of the rdc:

```
/
- chrom_sizes/
    - chrom | O
    - size | int64
- background
    - chrN
        - start | int64
        - end | int64
        - count | int64
- pixels
```

```
- rnaN
    - chrN
        - start | int64
        - end | int64
        - signal_count | float64
        - bg_count | float64
        - raw_bg_prob | float64
        - scaling_factor | float64
        - bg_prob | float64
        - impute | bool
        - signal_prob | float64
        - fc | float64
        - pvalue | float64
        - qvalue | float64
        - qvalue_global | float 64 | in v1.1
        - qvalue_rna | float64 | in v1.1
```

File-level attributes:

**Field**	**Type**	**Description**
is_scaling_fitted	bool	Whether scaling is estimated and RNAs background levels of interactions are rescaled
are_peaks_estimated	bool	Whether p-values and q-values for peaks are estimated
version	str	rdc schema version.<br>rdc schema version. Currently "1" and "1.1" are supported

RNA-level attributes contain source gene coordinates, contact statistics, binning, and spline parameters:

Field	Type	Description
chrom	O	RNA gene chromosome
start	int64	RNA gene start
end	int64	RNA gene end
total_contacts	int64	Total number of RNA contacts
genic_contacts	int64	Number of RNA contacts inside its gene
*cis*_contacts	int64	Number of *cis* RNA contacts: on the RNA's origin chromosome but outside RNA gene
*trans*_contacts	int64	Number of *trans* RNA contacts: on all chromosomes except for RNA's origin one
eligible	bool	Whether this RNA has enough contacts for further processing
*cis*_factor	float64	*cis* factor value for binning
*cis*_start	int64	Initial *cis* bin size
*trans*_bin_size	int64	*trans* bin size
scaling_spline_t	Float array	A vector of knots of a scaling B-spline
scaling_spline_c	Float array	A vector of scaling B-spline coefficients
scaling_spline_k	int64	A degree of a scaling B-spline

4.3 Default parameter values

Parameter	Meaning	Default	Sensible range	Tradeoff
**Bin size selection parameters**				
mcon, min_contacts	Minimal number of contacts to consider an RNA	1000	500-5000	Between sensitivity (lower) and specificity (higher)
tmin, trans_min	Minimal *trans* bin size, nt	1000	500-10000	Between precision (lower) and statistical power (higher)
tmax, trans_max	Maximal *trans* bin size, nt	1000000	100000-2000000	Between precision (lower) and performance (higher)
tstep, trans_step	Step for increasing the *trans* bin size, nt	1000	500-5000	Between precision (lower) and performance (higher)

cmin, cis_min	Minimal cis factor	1.1	1.01-1.5	How long proximal cis interactions are considered as cis and not *trans* interactions.
cmax, cis_max	Maximal cis factor	2.0	1.5-2.0	How long proximal cis interactions are considered as cis and not *trans* interactions.
cstep, cis_step	Step for increasing the cis factor	0.01	0.01-0.1	Between precision (lower) and performance (higher)
cstart, cis_start	The size of the starting cis bin	5000	1000-10000	Should be selected based on the *trans* bin size parameters.
tol, tolerance	Maximal absolute difference between two consecutive cost function values to consider optimization converged	0.01	0.01-0.1	Between precision of convergence (lower) and effective dealing with noise (higher)
w, window	Window size to average cost function values over	1	1-20	Between precision of the selection procedure (lower) and effective dealing with noise (higher)
**Background generation parameters**				
bs, binsize	The bin size of the generated background track	1000	500-10000	Between precision (lower) and accounting for local effects, such as copy numbers (higher)
bt, bgtype	The type of background to construct	rnas	rnas, custom, uniform	If rnas, will take *trans* contacts of the supplied list of RNAs (used for ATA data). If custom, will take the supplied bedGraph file (used for OTA data). If uniform, will create a uniform background (in case the user does not want to model the chromatin heterogeneity).
**RDC creation parameters**				

i, ifactor	Imputation factor for zero background coverage: i times average background coverage	0.01	0.001-0.1	Between type I (lower) and type II (higher) errors
d, degree	Spline degree	3	3	Not tested for other degrees
mt, max_threshold	Maximal binomial p-value to consider a point an outlier in a spline refinement procedure.	0.05	0.01-0.1	Between stringency of filtering (lower) and statistical power (higher)
nr, no_refine	If included, do not apply a spline refinement procedure	False	False, True	If included, might lead to underestimation of peaks
fv, fill_value	Fold-change fill ratio in case of 0/0 between signal and background	1	1	NA
**Peak filtering parameters**				
q, qval_threshold	Q-value threshold for peaks	0.05	0.1-0.001	Between statistical precision (lower) and number of peaks (higher)
qt, qval_type	Q-value type to use for thresholding peaks	global	global, rna	Global q-values are estimated over the whole dataset, while RNA q-values are estimated within each RNA separately. The choice depends on how one wants to approach the multiple-testing correction.
**Processing**				
c, cores	Number of cores to parallelize the algorithm	1	1-20	Between low memory footprint (lower) and speed (higher)

II. RNA-DNA contact data simulation framework

1 Definitions

We simulate contacts of a single RNA. Based on the BaRDIC model of non-specific RNA-DNA contacts, the probability of a non-specific contact of this RNA with a point x on the genome from a Cr_x chromosome is proportional to

$$p(x|Cr_x = Cr_{RNA}) \propto p_{cis} \cdot p_{scaling}(d) \cdot p_{Bg}(x),$$

$$p(x|Cr_x \neq Cr_{RNA}) \propto p_{trans} \cdot p_{Bg}(x),$$

where $p_{Bg}(x)$ models the genomic background at the point x, which reflects chromatin heterogeneity and the density of restriction sites, $p_{scaling}(d)$ models an increase of observed contact frequencies in cis compared to the background distribution at distance d from the source RNA gene, and p_{cis} and p_{trans} are equal to fractions of *cis* and *trans* contacts, respectively.

Specific contacts corresponding to peaks are modelled as additional contacts.

2 Modeling procedure

2.1 Chromosomes

We model two chromosomes: *cis* and *trans*. The total length of the genome and the length of the *cis* chromosome are supplied by a user. Estimated chromosome sizes are saved in a tab-separated file.

2.2 Binning

Two chromosomes are binned with *bioframe.binnify* into equally sized bins of the supplied size. These bins are later used for creating histograms to sample the coordinates of non-specific and specific contacts.

2.3 Background

Background counts for genomic bins are sampled from the Poisson distribution (*scipy.stats.pois*) with parameter λ supplied by the user, which equals the average background value per bin.

We use the Poisson distribution due to its simplicity and availability in scipy. However, it doesn't take into account multiple features of the real background (Note Figure 3A):

1. Inflation of zero values (telomeres, centromeres, and dropout).
2. Overdispersion of background values (non-uniformity of the background).
3. Heavy right tail of the distribution (highly accessible and/or repetitive regions).

Due to that, the simulated background is not as heterogeneous as the real one (Note Figure 3B), so estimating its heterogeneity with BaRDIC didn't improve peak calling results much compared to the uniform background setting.

The simulated background track is saved as a bedGraph file.

2.4 Source gene coordinates

For the sake of simulations, we assume the source gene can be modelled as a single point on the *cis* chromosome. The coordinate of the gene point is sampled uniformly in a region of the *cis* chromosome bound by B_{left} and B_{right} – fractions of the *cis* chromosome length to mimic the telomeres. By default, B_{left} is 0.05, and B_{right} is 0.95. The source gene coordinates are saved as a gene annotation file in BED format.

2.5 RD-scaling

RD-scaling is modelled in the log-log space to mimic the shape of real RD-scaling curves. By definition,

$$\lg(p_{scaling}(d)) \equiv \lg \frac{N_{cis}(d)}{p_{bg}(d)} = f(\lg d).$$

Function f is modelled using a sigmoid function:

$$\lg d = D, f(D) = \frac{A}{1 + e^(B + C \cdot D)},$$

where A, B, and C are parameters.

This function has two limits:

1. $\lim_{D \to -\infty} f(D) = A$, hence A – is the maximal RD-scaling value, log fold-change of the observed *cis* contact density over the background contact density near the source gene. This value is supplied by the user.

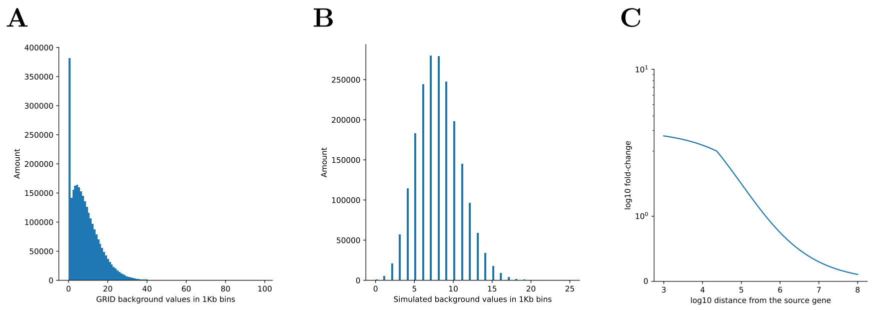

Note Figure 3: Distributions for simulations. **(A)** Distribution of background values for GRID, estimated with BaRDIC. **(B)** Simulated distribution of background values. **(C)** Sigmoid function profile for values $A = 3, D_{high} = 3, F_{high} = 0.9, D_{low} = 7, F_{low} = 0.1$.

2. $\lim_{D \to +\infty} f(D) = 0$, i.e. the frequency of non-specific *cis*-contacts is not different from the background frequency at large distances from the source gene. This behaviour is in line with the one of the real RD-scaling.

We estimate parameters B and C indirectly by asking a user to supply four additional values, D_{high}, F_{high}, D_{low}, and F_{low}. F_{low} and F_{high} are fractions of the maximal RD-scaling A andD_{low} and D_{high} are log-separations they are achieved at. These values are bound by the following formulae:

- $f(D_{high}) = F_{high} \cdot A$,
- $f(D_{low}) = F_{low} \cdot A$,
- $0 < F_{low} < F_{high} < 1$,
- $D_{low} > D_{high}$.

Values of B and C can be estimated by substituting D and f(D) in the sigmoid equation with the presented values and solving the resulting system of linear equations (e.g., with Kramer's rule):

$$B = \frac{D_{low} + D_{high}}{D_{low} - D_{high}} \cdot \ln \frac{F_{low}}{F_{high}}, C = \frac{2}{D_{low} - D_{high}} \cdot \ln \frac{F_{high}}{F_{low}}$$

Default values: F_{high} is 0.9 and D_{high} is 3, which corresponds to RD-scaling being 90% of its max value at 1Kb apart from the source gene; F_{low} is 0.1 and D_{low} is 7, which corresponds to RD-scaling being 10% of its max value at 10Mb apart from the source gene (Note Figure 3C).

2.6 Simulating non-specific contacts

The total amount of non-specific contacts and the fraction of *cis* contacts within them are supplied by the user. Coordinates are sampled from a scaling-corrected background distribution, which is constructed in several steps:

1. Zero values in the background are imputed with a very low value (by default 1% of the average background value per bin) to allow for rare samplings of contact coordinates from these zero bins.

2. Scaling factors for *cis*-chromosomal bins are estimated by calculating the relative distances of those bins to the source gene point, supplying logarithms of those distances into the RD-scaling sigmoid function, and taking 10 to powers of the resulting numbers.

   (a) For each bin, there are three distances to the source gene point: from the bin start, the bin end, and the bin midpoint. Scaling factors are estimated for each of the three distances and then averaged before taking 10 to their powers.

3. Background counts of *cis*-chromosomal bins are multiplied by the obtained scaling factors.

4. *Cis* and *trans*-chromosomal backgrounds are split and their counts are normalized to sum to 1 separately.

5. *Cis* and *trans*-chromosomal backgrounds are fitted with *scipy.stats.rv_histogram*.

Coordinates of non-specific contacts are sampled from these two histograms in a proportion of *cis* and *trans* contacts defined by a user. These coordinates are written to a BED file.

2.7 Simulating locations of peaks and specific contacts

The number of peaks and the fraction of *cis* peaks are supplied by the user. Coordinates of peak summits are sampled again from scaling-corrected background distributions separately for the *cis* and the *trans* chromosomes.

The amounts of additional specific contacts for each peak are sampled from the Poisson distribution with the λ parameter representing the average number of specific contacts per peak, which is supplied by a user.

Coordinates of additional specific contacts for each peak are sampled from the normal distribution with a mean equal to the peak summit coordinate and standard deviation equal to a supplied number of nts.

Coordinates of peak summits with corresponding amounts of additional specific contacts are saved to a BED file. Coordinates of all specific contacts (including peak summits) are saved to another BED file. Coordinates of all contacts (both specific and non-specific) are saved to yet another BED file.

3 Output files

1. chrom.sizes – sizes of the *cis* and the *trans* chromosome (UCSC-like format).

2. annot.bed – coordinates of the source gene of the simulated RNA.

3. background.bedGraph – the simulated background track.
4. non_specific_contacts.bed – coordinates of non-specific contacts.
5. peak_summits.bed – coordinates of peak summits with corresponding amounts of specific contacts.
6. specific_contacts.bed – coordinates of specific contacts.
7. contacts.bed – coordinates of all contacts.

Output files chrom.sizes, annot.bed, background.bedGraph, contacts.bed can be directly submitted to BaRDIC with the option -bt custom.

4 Simulation parameters

**Parameter**	**Description**	**Values used in this study**
L_cis	The length of the *cis* chromosome	1e8 (100Mb)
L_genome	The total length of the genome	2e9 (2Gb)
bin_size	Background bin size	1e3 (1Kb)
bg_exp	The average value in a single background bin (Poisson lambda)	8
imputation_factor	Factor times bg_exp to impute zero counts for simulation of contacts	0.01
A	MaxRD-scaling value (log fold-change) at the gene point	3
D_high	lg distance of F_high times A	3
D_low	lg distance of F_low times A	7
F_high	Fraction of the maximal RD-scaling A achieved at lg distance D_high	0.9
F_low	Fraction of the maximal RD-scaling A achieved at lg distance D_low	0.1
B_left	Start of the region on the *cis* chromosome to sample the gene point from (as a fraction of the chromosome length)	0.05
B_right	End of the region on the *cis* chromosome to sample the gene point from (as a fraction of the chromosome length)	0.95
N_total	Total number of simulated non-specific contacts	20000
frac_cis	Fraction of simulated non-specific contacts in *cis*	0.25
N_peaks	Number of simulated peaks	20
frac_cis_peaks	Fraction of peaks in *cis*	0.5
peak_exp	Expected number of contacts per peak (Poisson lambda)	10
sd	Standard deviation of the peak contacts positions (Normal sigma)	3000

References

[1] Yoav Benjamini, Dan Drai, Greg Elmer, Neri Kafkafi, and Ilan Golani. Controlling the false discovery rate in behavior genetics research. *Behavioural Brain Research*, 125(1-2):279–284, November 2001.

[2] Qiang Song and Andrew D. Smith. Identifying dispersed epigenomic domains from ChIP-seq data. *Bioinformatics*, 27(6):870–871, February 2011.

[3] Mahmoud M. Ibrahim, Scott A. Lacadie, and Uwe Ohler. JAMM: a peak finder for joint analysis of NGS replicates. *Bioinformatics*, 31(1):48–55,

September 2014.

[4] Hamid Alinejad-Rokny, Rassa Ghavami Modegh, Hamid R. Rabiee, Ehsan Ramezani Sarbandi, Narges Rezaie, Kin Tung Tam, and Alistair R. R. Forrest. MaxHiC: A robust background correction model to identify biologically relevant chromatin interactions in hi-c and capture hi-c experiments. *PLOS Computational Biology*, 18(6):e1010241, June 2022.

[5] Arya Kaul, Sourya Bhattacharyya, and Ferhat Ay. Identifying statistically significant chromatin contacts from hi-c data with FitHiC2. *Nature Protocols*, 15(3):991–1012, January 2020.

[6] Yong Zhang, Tao Liu, Clifford A Meyer, Jérôme Eeckhoute, David S Johnson, Bradley E Bernstein, Chad Nusbaum, Richard M Myers, Myles Brown, Wei Li, and X Shirley Liu. Model-based analysis of ChIP-seq (MACS). *Genome Biology*, 9(9), September 2008.

[7] Mark Carty, Lee Zamparo, Merve Sahin, Alvaro González, Raphael Pelossof, Olivier Elemento, and Christina S. Leslie. An integrated model for detecting significant chromatin interactions from high-resolution hi-c data. *Nature Communications*, 8(1), May 2017.

[8] Guido Van Rossum and Fred L. Drake. *Python 3 Reference Manual*. CreateSpace, Scotts Valley, CA, 2009.

[9] Charles R. Harris, K. Jarrod Millman, Stéfan J. van der Walt, Ralf Gommers, Pauli Virtanen, David Cournapeau, Eric Wieser, Julian Taylor, Sebastian Berg, Nathaniel J. Smith, Robert Kern, Matti Picus, Stephan Hoyer, Marten H. van Kerkwijk, Matthew Brett, Allan Haldane, Jaime Fernández del Río, Mark Wiebe, Pearu Peterson, Pierre Gérard-Marchant, Kevin Sheppard, Tyler Reddy, Warren Weckesser, Hameer Abbasi, Christoph Gohlke, and Travis E. Oliphant. Array programming with NumPy. *Nature*, 585(7825):357–362, September 2020.

[10] Jeff Reback, Wes McKinney, jbrockmendel, Joris Van den Bossche, Tom Augspurger, Phillip Cloud, gfyoung, Sinhrks, Adam Klein, Matthew Roeschke, Jeff Tratner, Chang She, William Ayd, Simon Hawkins, Terji Petersen, Jeremy Schendel, Andy Hayden, Marc Garcia, Vytautas Jancauskas, MomIsBestFriend, Pietro Battiston, Skipper Seabold, chris b1, h vetinari, Stephan Hoyer, Wouter Overmeire, alimcmaster1, Mortada Mehyar, Christopher Whelan, and Thomas Kluyver. pandas-dev/pandas: Pandas 1.0.0, January 2020.

[11] Pauli Virtanen, Ralf Gommers, Travis E. Oliphant, Matt Haberland, Tyler Reddy, David Cournapeau, Evgeni Burovski, Pearu Peterson, Warren Weckesser, Jonathan Bright, Stéfan J. van der Walt, Matthew Brett, Joshua Wilson, K. Jarrod Millman, Nikolay Mayorov, Andrew R. J. Nelson, Eric Jones, Robert Kern, Eric Larson, C J Carey, İlhan Polat, Yu Feng, Eric W. Moore, Jake VanderPlas, Denis Laxalde, Josef Perktold, Robert

Cimrman, Ian Henriksen, E. A. Quintero, Charles R. Harris, Anne M. Archibald, Antônio H. Ribeiro, Fabian Pedregosa, Paul van Mulbregt, Aditya Vijaykumar, Alessandro Pietro Bardelli, Alex Rothberg, Andreas Hilboll, Andreas Kloeckner, Anthony Scopatz, Antony Lee, Ariel Rokem, C. Nathan Woods, Chad Fulton, Charles Masson, Christian Häggström, Clark Fitzgerald, David A. Nicholson, David R. Hagen, Dmitrii V. Pasechnik, Emanuele Olivetti, Eric Martin, Eric Wieser, Fabrice Silva, Felix Lenders, Florian Wilhelm, G. Young, Gavin A. Price, Gert-Ludwig Ingold, Gregory E. Allen, Gregory R. Lee, Hervé Audren, Irvin Probst, Jörg P. Dietrich, Jacob Silterra, James T Webber, Janko Slavič, Joel Nothman, Johannes Buchner, Johannes Kulick, Johannes L. Schönberger, José Vinícius de Miranda Cardoso, Joscha Reimer, Joseph Harrington, Juan Luis Cano Rodríguez, Juan Nunez-Iglesias, Justin Kuczynski, Kevin Tritz, Martin Thoma, Matthew Newville, Matthias Kümmerer, Maximilian Bolingbroke, Michael Tartre, Mikhail Pak, Nathaniel J. Smith, Nikolai Nowaczyk, Nikolay Shebanov, Oleksandr Pavlyk, Per A. Brodtkorb, Perry Lee, Robert T. McGibbon, Roman Feldbauer, Sam Lewis, Sam Tygier, Scott Sievert, Sebastiano Vigna, Stefan Peterson, Surhud More, Tadeusz Pudlik, Takuya Oshima, Thomas J. Pingel, Thomas P. Robitaille, Thomas Spura, Thouis R. Jones, Tim Cera, Tim Leslie, Tiziano Zito, Tom Krauss, Utkarsh Upadhyay, Yaroslav O. Halchenko, and Yoshiki Vázquez-Baeza and. SciPy 1.0: fundamental algorithms for scientific computing in python. *Nature Methods*, 17(3):261–272, February 2020.

[12] Skipper Seabold and Josef Perktold. Statsmodels: Econometric and statistical modeling with python. In *Proceedings of the Python in Science Conference*. SciPy, 2010.

[13] Open2C, Nezar Abdennur, Geoffrey Fudenberg, Ilya Flyamer, Aleksandra A. Galitsyna, Anton Goloborodko, Maxim Imakaev, and Sergey V. Venev. Bioframe: Operations on genomic intervals in pandas dataframes. February 2022.

[14] The HDF Group. Hierarchical data format version 5, 2000-2010.

[15] Casper da Costa-Luis, Stephen Karl Larroque, Kyle Altendorf, Hadrien Mary, richardsheridan, Mikhail Korobov, Noam Yorav-Raphael, Ivan Ivanov, Marcel Bargull, Nishant Rodrigues, Guangshuo Chen, Antony Lee, Charles Newey, CrazyPython, JC, Martin Zugnoni, Matthew D. Pagel, mjstevens777, Mikhail Dektyarev, Alex Rothberg, Alexander Plavin, Fabian Dill, FichteFoll, Gregor Sturm, HeoHeo, Hugo van Kemenade, Jack McCracken, MapleCCC, Max Nordlund, and Mike Boyle. tqdm: A fast, Extensible Progress Bar for Python and CLI, August 2023.
